# Efficacy of FiberMore, an AI-Based mHealth Intervention to Increase Dietary Fiber Intake Among Type 2 Diabetes Patients: Protocol for a Pilot Randomized Controlled Trial

**DOI:** 10.2196/78019

**Published:** 2025-12-04

**Authors:** Wei Thing Sze, Kayo Waki, Daniel Lane, Kyohei Hasegawa, Ryohei Nakada, Shuya Iwata, Yuexiang Ji, Akihiro Isogawa, Tomohisa Aoyama, Kana Miyake, Yuri Kadowaki, Tomoya Kawaguchi, Yoshinori Matsuo, Kengo Miyoshi, Nagisa Ishibashi, Gotaro Toda, Saori Kameda, Masaki Igarashi, Masaki Tanaka, Toshimasa Yamauchi, Masaomi Nangaku

**Affiliations:** 1 Department of Clinical Information Engineering, Graduate School of Medicine, The University of Tokyo, 7 Chome-3-1 Hongo, Bunkyo City, Tokyo, 113-8654, Japan, 81-3-58411892; 2 Department of Planning, Information and Management, The University of Tokyo Hospital, Tokyo, Japan; 3 Department of Health Sciences and Nursing (Health Informatics), Graduate School of Medicine, The University of Tokyo, Tokyo, Japan; 4 Department of Diabetes and Metabolic Diseases, The University of Tokyo, 7 Chome-3-1 Hongo, Bunkyo City, Tokyo, 113-8654, Japan, 81 03-3812-2111; 5 Division of Diabetes, Mitsui Memorial Hospital, Tokyo, Japan; 6 Division of Nephrology and Endocrinology, Graduate School of Medicine, The University of Tokyo, Tokyo, Japan

**Keywords:** digital therapeutics, behavior change, theory of planned behavior, dietary fiber, artificial intelligence, type 2 diabetes, randomized controlled trial

## Abstract

**Background:**

A high intake of dietary fiber has been shown to improve glycemic control and decrease hyperinsulinemia in people living with type 2 diabetes (T2D). T2D patients in Japan consume less than the recommended amount of fiber. Based on findings from a formative study, we developed an artificial intelligence (AI)-powered mobile health (mHealth) intervention, FiberMore, that uses the theory of planned behavior to help T2D patients increase their dietary fiber intake by enhancing their perceived behavioral control and attitude toward fiber consumption.

**Objective:**

We aimed to assess the efficacy of FiberMore in improving the dietary fiber intake of T2D patients by conducting a pilot randomized controlled trial. In addition, we want to explore the efficacy of FiberMore in reducing HbA_1c_ of T2D patients via improvement in dietary fiber intake.

**Methods:**

This is a randomized, single-blinded, multicenter study targeting 80 T2D patients from 3 institutions in Japan with a 2-week run-in, a 12-week intervention, and a 12-week observation. The intervention group is given access to FiberMore throughout the 12-week intervention period. A core feature of FiberMore is AI-powered meal photo logging using a fine-tuned GPT-4o (OpenAI) model, which analyzes the nutrient content of meals and delivers personalized, real-time feedback on fiber content. In addition, FiberMore provides personalized fiber goal setting and supports participants in identifying barriers to increasing fiber intake, along with corresponding coping strategies (labeled as “solutions” to the participant), through an AI chatbot. The AI chatbot also assesses participants’ emotional attitudes toward eating more fiber and delivers relevant educational content on dietary fiber. The control group receives a sham intervention focused on salt reduction, consisting of educational content delivered at 3 time points during the intervention period and records their daily efforts in salt reduction in a diary. The 12-week intervention period will be followed by a 12-week observational period to investigate the sustainability of the intervention’s effects. The primary outcome is between-group difference in the change of dietary fiber intake at 12 weeks. The secondary outcomes include HbA1c, other clinical measures, measurements of behavior changes, and assessment of participants’ satisfaction and perceived usefulness of the intervention.

**Results:**

Recruitment began on February 12, 2025, and ended on September 1, 2025. We anticipate that the intervention period will conclude in December 2025 and the observation period will conclude in March 2026. As of September 22, 2025, a total of 72 participants have been officially enrolled and randomized.

**Conclusions:**

There are currently no mHealth dietary interventions that specifically focus on increasing fiber intake in Japan, highlighting the novelty of this intervention. This trial will generate important evidence on the efficacy, feasibility, and safety of an AI-based mHealth intervention for enhancing dietary fiber intake and glycemic control in free-living individuals with T2D. Furthermore, as a pilot study, it will offer valuable insights into the development of AI as a promising tool for accurate, low-burden dietary assessment.

## Introduction

Globally, approximately 462 million individuals are affected by type 2 diabetes (T2D) [1]. In Japan, the Ministry of Health, Labor, and Welfare estimated in 2019 that 11 million people are suspected of having diabetes, with a 19.7% prevalence in males and 10.8% in women [2]. T2D is associated with a variety of medical complications, including kidney failure, coronary heart disease, and stroke [3].

Dietary fiber is a macronutrient of importance in the nutrition management of diabetes patients [4]. A high intake of dietary fiber has been shown to improve glycemic control and decrease hyperinsulinemia in people living with T2D [5]. The impact of higher fiber content on reducing the glycemic index of foods has been a long-standing focus in diabetes treatment research [6]. Several meta-analyses have consistently demonstrated that increased dietary fiber intake leads to significant improvements in glycemic control, with reductions in HbA_1c_ of at least 0.3% [7-11].

Other benefits of high intake of dietary fiber include weight management, lowering of lipid levels, and lowering of blood pressure [12,13]. Additionally, T2D patients consuming more than 25 g of total dietary fiber per day had a notably low incidence rate of stroke (<0.90 per 1000 patient-years) [14]. In view of the benefits of increased dietary fiber intake, several clinical guidelines recommend daily fiber targets for individuals with diabetes: the Japan Diabetes Society (JDS) advises at least 20 g per day [15]; the European Association for the Study of Diabetes recommends 35 g or more per day [16]; and the American Diabetes Association suggests 25‐38 g per day or a minimum of 14 g per 1000 kcal [17]. Nevertheless, T2D patients in Japan consume less than the recommended intake. In a cross-sectional study of 4399 Japanese patients with T2D, the average dietary fiber intake was about 13 g per day [18]. In addition, a cohort study of 1414 Japanese patients with T2D reported that approximately 75% of the cohort consumed less than 16 g of dietary fiber per day [14].

Mobile health (mHealth) lifestyle self-management interventions, typically targeting diet and physical activity, have been proven effective in achieving clinically meaningful glycemic control among T2D patients [19-21]. mHealth apps that support diet self-management improve diet quality and enhance adherence to self-monitoring more effectively than traditional pen-and-paper methods [22,23]. mHealth offers advantages such as delivering interventions anytime and anywhere, supporting extended engagement, and enabling personalized communication [24]. Nevertheless, previous research suggested that Japanese T2D patients who monitored their diet using an mHealth diabetes self-management app consumed less than the recommended 20 g of dietary fiber per day, despite engaging in self-monitoring through meal logging and receiving general nutritional feedback including dietary fiber [25]. This suggests a general diet management app is insufficient to help T2D patients increase their fiber intake. Additionally, many existing nutritional management apps are not grounded in behavior change theory [26], which hampers the understanding of the mechanisms through which the apps influence behavior [27]. Interventions based on behavior change theory have been shown to be more effective than non–theory-based interventions [28], with those using the theory of planned behavior (TPB) [29] being especially effective in understanding diet behaviors [30]. The inclusion of behavior change theory in mHealth apps has also been reported to enhance user engagement [31]. Given the well-established benefits of dietary fiber in glycemic control for T2D patients, there is a clear need for a fiber-focused mHealth dietary intervention among this population. Previous research has demonstrated that such interventions are effective in increasing fiber consumption [32-34].

A recent study based on the TPB revealed that while T2D patients in Japan are aware of the benefits of consuming more dietary fiber, the 2 main barriers they faced are a lack of knowledge about high-fiber foods and difficulty in estimating fiber content in meals [35]. Attitude and perceived behavioral control (PBC) were found to predict intention to increase 50% more dietary fiber than patients’ usual intake [35]. This highlights the need for an intervention that provides real-time feedback on dietary fiber intake for each meal, along with educational information to support fiber consumption. Nevertheless, evidence has suggested that manual food logging in dietary apps can be burdensome, leading to reduced user engagement in diet self-monitoring over time [19,26]. Image-based dietary assessment, facilitated by artificial intelligence (AI), could address this issue by streamlining the process of food logging, thus reducing user burden [36]. This method has also been shown to potentially exceed the accuracy of human estimations of nutrient content based on digital food images [37], particularly in the assessment of dietary fiber [38,39]. Furthermore, AI is capable of offering personalized nutritional advice and meal plans [40]. Leveraging this strength of AI allows us to build a diet intervention in line with the JDS’s recommendation to honor patients’ personal diet preferences while administering medical nutrition therapy [15].

Based on the above research findings, we developed FiberMore, an AI-powered diet intervention app that uses TPB to help T2D patients increase their dietary fiber intake by enhancing their PBC and attitude toward fiber consumption. To our knowledge, this is the first study to use an AI-based bot to deliver fiber-focused feedback based on the assessment of meal photos. While many nutrition apps focus on improving general health care concerns, apps that focus on specific chronic diseases and the nutrition involved in the disease state are limited [26]. Currently, dietary interventions specifically targeting improving dietary fiber intake among T2D patients have not been developed in Japan, thus highlighting the novelty and importance of this behavior change intervention. The purpose of this single-blind, randomized controlled trial is to examine the preliminary efficacy of an AI-assisted, app-based mHealth intervention to promote dietary fiber intake among T2D patients, leading to improved blood glucose control.

The specific research objectives and hypotheses are as follows:

1. Objective 1: To investigate the efficacy of FiberMore in improving dietary fiber intake.
  - We hypothesize that, at the end of the intervention, the intervention group will achieve statistically significant improvement in the primary outcome, dietary fiber intake, relative to the dietary fiber intake of the control group.
2. Objective 2: To investigate the preliminary efficacy of FiberMore in reducing HbA_1c_ levels.
  - We hypothesize that, at the end of the intervention, the intervention group will achieve a statistically significant reduction in the secondary outcome, HbA_1c_ levels, relative to HbA_1c_ levels in the control group.

## Methods

### Study Overview

**Figure 1.**
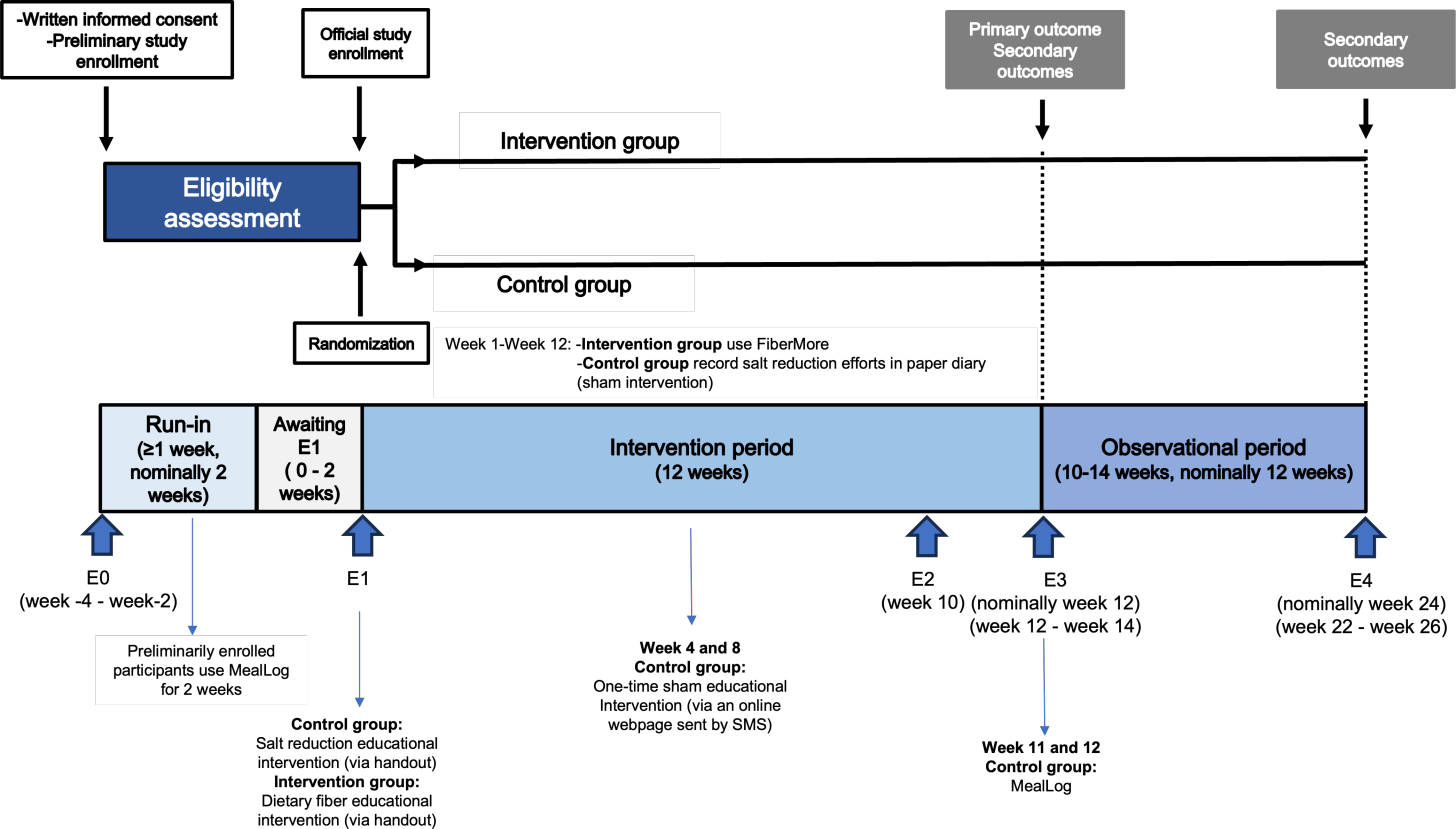
Overview of study timeline.

This is a single-blind, parallel-group pilot randomized controlled trial targeting patients with T2D who consume less than 20 g of dietary fiber per day on average. Following a 2-week run-in period, study participants will undergo a 12-week intervention period, followed by a 12-week observation period, making the total study duration 26 weeks (with a maximum of 30 weeks, including allowable ranges in clinic visits; see Figure 1).

The intervention group is given access to an AI-powered dietary fiber intake support system named “FiberMore” throughout the 12-week intervention period. The intervention offers several features, including fiber content assessment of meals through meal photos and interacting with a chatbot, tailored feedback on logged meals, personalized fiber goal setting, barrier identification to eat more fiber and strategies (solutions) to overcome them, emotional attitude assessment toward eating more fiber, and access to educational information on dietary fiber via a chatbot.

The study will use a “sham intervention” related to dietary behavior change as control [41]. The main purpose of using a sham intervention is to achieve participant blinding and to control for the Hawthorne effect of receiving an intervention [42]. Salt reduction education aligns with dietary recommendation for T2D management. The educational content specifically focuses on reducing sodium intake through cooking methods and seasoning practices, without targeting food choices related to dietary fiber. The control group receives educational intervention on salt reduction at 3 time points: at official study enrollment (via an educational handout), at week 4 (through web-based educational information delivered via SMS), and at week 8 (through web-based educational information delivered via SMS). Additionally, participants in the control group will document their salt reduction efforts in a paper diary throughout the intervention period. They will use the meal logging app “MealLog” to record their meals at week 11 and 12, which allows for meal logging without providing any feedback or dietary intervention.

### Setting and Participants

Patients are recruited at The University of Tokyo Hospital and Mitsui Memorial Hospital, both located in central Tokyo. Recruitment will be conducted by attending physicians during patients’ regular consultations at outpatient clinics. In order to select patients who will benefit from the interventions in this study and can appropriately participate in this study, we apply the inclusion criteria (see Textbox 1). In order to exclude patients whose factors may affect the evaluation of this study’s efficacy, and to ensure the intervention is safe for the participants, we apply the exclusion criteria (Textbox 2). Most of the criteria apply on the day of obtaining consent (at the E0 event), with some applying before randomization at the E1 event, based on results from the run-in period.

Textbox 1. **Inclusion criteria of the study.**

At the time of consent acquisition (E0):

- Diagnosed with type 2 diabetes (T2D) and attending the hospital for T2D treatment
- HbA_1c_ 7.5% and above and owns a smartphone with internet data connection (either an iPhone or an Android phone) and are able to receive SMS text messages
- Willing to take all meal photos with their smartphones and log them on an app
- Patients are able to attend research appointments at designated times during the research period
- Aged 18 years and older
- Fluent in spoken and written Japanese
- In the contemplation, preparation, and action stage to increase dietary fiber intake according to the transtheoretical model (TTM) of behavior change
- Determined by the recruiting researcher to have no cognitive impairment
- Determined by the recruiting researcher to be capable of receiving dietary fiber intervention
- Patients have received a thorough explanation about participation in this study, participants have provided voluntary, written consent based on a complete understanding of the study.

Textbox 2. **Exclusion criteria of the study.**

At the time of consent acquisition (E0):

- Aged 76 years and older
- Judged by physicians to be an unsuitable candidate for dietary intervention
- Changed type 2 diabetes (T2D) medication within the past 8 weeks
- Currently participating in another clinical research program
- Undergoing a fiber-restricted diet as ordered by physicians
- History of eating disorder
- Currently taking fiber supplements
- Patients with fluid restriction
- Patients with Crohn disease, ulcerative colitis, irritable bowel disease, irritable bowel syndrome, and intestinal obstruction
- Patients who have taken bowel surgery within the last 3 months
- Patients with chronic kidney disease stage 4 and above
- Patients with hyperkalemia or hyponatremia
- Patients who are currently pregnant or actively trying to conceive
- Patients whose smartphones are incompatible with the app, either due to the inability to download or run it effectively, including smartphones with operating systems below Android 5.0 and iOS 14.0
- Any other reason determined by the physician that deems the patient unfit for participation

Before group allocation (after the run-in, before event E1):

- Takes a daily average of 20 g and above of dietary fiber, determined via app assessment over the 2 weeks run-in period
- Fail to log at least 2 eating occasions (either main meals or snacks) in the app per day for 7 or more days during the 2 weeks run-in period

### Intervention design

**Figure 2.**
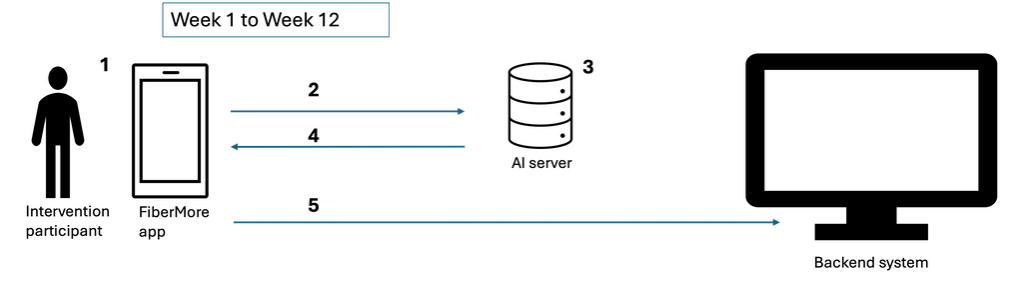
System and equipment used in the intervention group during the intervention period (week 1 to week 12). AI: artificial intelligence.

The AI-based dietary fiber intake support system for the intervention group consists of a smartphone app (iOS and Android) that delivered intervention features to promote dietary fiber intake, named “FiberMore.” An AI-powered chatbot, named “EiYouBot,” uses Anthropic’s Claude Sonnet model with FiberMore-specific prompts. The image processing uses OpenAI’s GPT-4o (1-shot prompting, with fine-tuning). These 2 AI approaches are integrated within FiberMore (see Figure 2). GPT-4o and Claude Sonnet are state-of-the-art large language models that have undergone extensive pretraining on diverse multimodal data. We previously reported methodology and findings of an internal validation study, where the FiberMore image analysis model was benchmarked against registered dietitians using Japanese meal photos. We found that the base AI model without fine-tuning demonstrated better accuracy in fiber estimation than a dietitian [38,39]. Building on the same validation methodology, we fine-tuned the AI model using a data set of over 1269 Japanese meals that included a combination of weighed food records and dietitian-estimated nutritional values. The final fine-tuned AI model used in this study demonstrated improved performance, with intraclass correlation coefficients (ICC) of 0.79 for fiber, surpassing the earlier non–fine-tuned models and the dietitian benchmark (ICC=0.68).

The FiberMore intervention design was guided by continuous, iterative discussions within the multidisciplinary research team, combined with repeated cycles of testing and refinement. The chatbot prompt was continuously improved through multiple rounds of testing with simulated and real-user inputs from individuals with T2D to ensure clarity, acceptability, and safety of the chatbot. The detailed full intervention features of FiberMore are listed in Table 1. Although FiberMore supports both Japanese and English, only the Japanese version will be used in this study.

**Table 1.** Details of FiberMore features used by the intervention group.

FiberMore features		Description
Daily intervention features		
Meal logging options in FiberMore		
Snap a meal (or upload from phone gallery)		Study participants log a meal via taking a photo with FiberMore. The meal photo is uploaded to the AI^a^ server and analyzed for its fiber, energy, carbohydrates, protein, fat, and salt amounts. If connectivity issues occur, study participants can take a meal photo using the native camera app of their smartphone and use the “Upload from gallery” function to upload the meal photo to FiberMore at a later time within the same day.
Chat a meal		Study participants describe meals with the “Chat a meal” chatbot (EiYouBot) on FiberMore to log their meals. Based on the descriptions, the meal is analyzed by the AI server for its fiber, energy, carbohydrates, protein, fat, and salt amounts.
Snap a meal (or upload from phone gallery) followed by chat a meal		Study participants first log a meal via taking a photo with FiberMore. The meal photo is uploaded to the AI server and analyzed for its fiber, energy, carbohydrates, protein, fat, and salt amount. Study participants then describe additional details about the meal with EiYouBot. Based on these extra details, the chatbot adjusts the estimates for the meal’s fiber, energy, carbohydrates, protein, fat, and salt amounts.
Real-time feedback on logged meals		For every meal logged onto FiberMore, study participants will receive 3 types of immediate feedback: Real-time line graph showing progress toward reaching their daily fiber goal. Feedback on the fiber amount of the meal in grams. Feedback on the amount of dietary fiber in a meal compared to the number of calories it provides, whether it is low, moderate, good, and excellent (Multimedia Appendix 1).^b^
Daily feedback on fiber goal achievement		Once a day, study participants will receive feedback on the app of whether they achieved the goal for fiber intake for the previous day. This feedback occurs via a pop-up screen when they open the app for the first time during the day.
Coping strategy (solution) assessment		Once a day, when study participants open the app for the first time, a pop-up screen will prompt them to assess whether they implemented their chosen solution for their selected barrier to increasing fiber intake on the previous day.
Weekly intervention features		
Goal setting		Once a week, study participants will be prompted by app notification from FiberMore to set a new weekly fiber goal. EiYouBot suggests a new goal based on the number of days the fiber goal is met in the previous week. Number of days fiber goal was met in the previous week: 0, 1, or 2 days: decrease 1 g from previous week’s goal. 3 or 4 days: maintain previous week’s goal. 5 days: increase 1 g from previous week’s goal. 6 or 7 days: increase 2 g from previous week’s goal. Study participants will respond in the chat with EiYouBot whether they are confident to meet the suggested goal at least 5 days in the week. If they are confident, the fiber goal suggested by the chatbot will be set as the new goal of the week. If study participants are not confident that they can meet the suggested goal, the chatbot will propose to lower the goal by 1 g, as long as it is not lower than the minimum goal of the study participants.^c^ Study participants can also negotiate with EiYouBot for an easier goal by proposing a lower goal in the chat. The proposed lower goal can be set as the new goal as long as it is not lower than the participant’s minimum goal.^c^ The maximum fiber goal that the chatbot can set is 30 g. The initial fiber goal for study participants assigned to the intervention group during official enrollment will be based on the 10th highest daily fiber intake logged during the 14-day run-in period, with no-log days considered as “0.” If the 10th highest value is 0, the smallest nonzero value logged during the run-in will be set as the initial goal.
Barrier and coping strategy setting		Once a week, study participants will be prompted by an app notification to choose a main barrier to increasing their fiber intake and determine a solution (ie, a coping strategy) to overcome that barrier via the “Barrier and Solution” chatbot. Study participants can select the barriers and solutions from the list provided by EiYouBot, or they can write their own barriers and solutions in the chat.
Emotional attitude assessment		Once a week, study participants will be prompted by an app notification to assess how they feel about increasing fiber intake via a chatbot, whether it is enjoyable, just okay, or not enjoyable. EiYouBot will provide positive and encouraging feedback based on the participant’s response.
On-demand feature		
Learn more		Study participants can use the “Learn more” chatbot anytime to receive educational information on dietary fiber by writing their questions to EiYouBot.

a AI: artificial intelligence.

b Based on the calculation of fiber to energy ratio of the meal eaten scaled to the daily fiber goal and target energy intake: low (<0.5), moderate (0.5‐0.89), good (0.9‐1.2), and excellent (>1.2). Refer to Multimedia Appendix 1 for calculation of target energy intake.

c The minimum fiber goal will be the same as the initial fiber goal of the study participants.

FiberMore is designed in line with the JDS’s guideline for T2D patients to consume 20 g and above of dietary fiber daily [15]. FiberMore uses intervention features such as goal setting, identifying barriers and coping strategies, and self-monitoring to help T2D patients reach this goal gradually. Furthermore, FiberMore uses AI to provide personalized feedback and advice for patients in line with JDS’s recommendation to consider patients’ personal dietary preferences when administering medical nutrition therapy [15].

The flow of interaction between the intervention participant, the app interface, the backend system, and the AI server is as follows, as illustrated in Figure 2: the intervention participant uploads a meal photo or engages with the chatbot feature in FiberMore (Step 1). Data provided by the participant is sent to the AI servers (Step 2). The AI servers process the uploaded meal photo or chatbot interaction and generate relevant feedback. Once the AI server completes processing, FiberMore receives relevant feedback by AI. This feedback is displayed to the user in real time within the app interface (Step 4). The app then securely transmits the feedback to the backend system via internet connection using a highly encrypted application programming interface (API) stored within the app (Step 5).

Description and app screenshots of participant interactions with FiberMore can be found in Multimedia Appendix 2.

**Figure 3.**
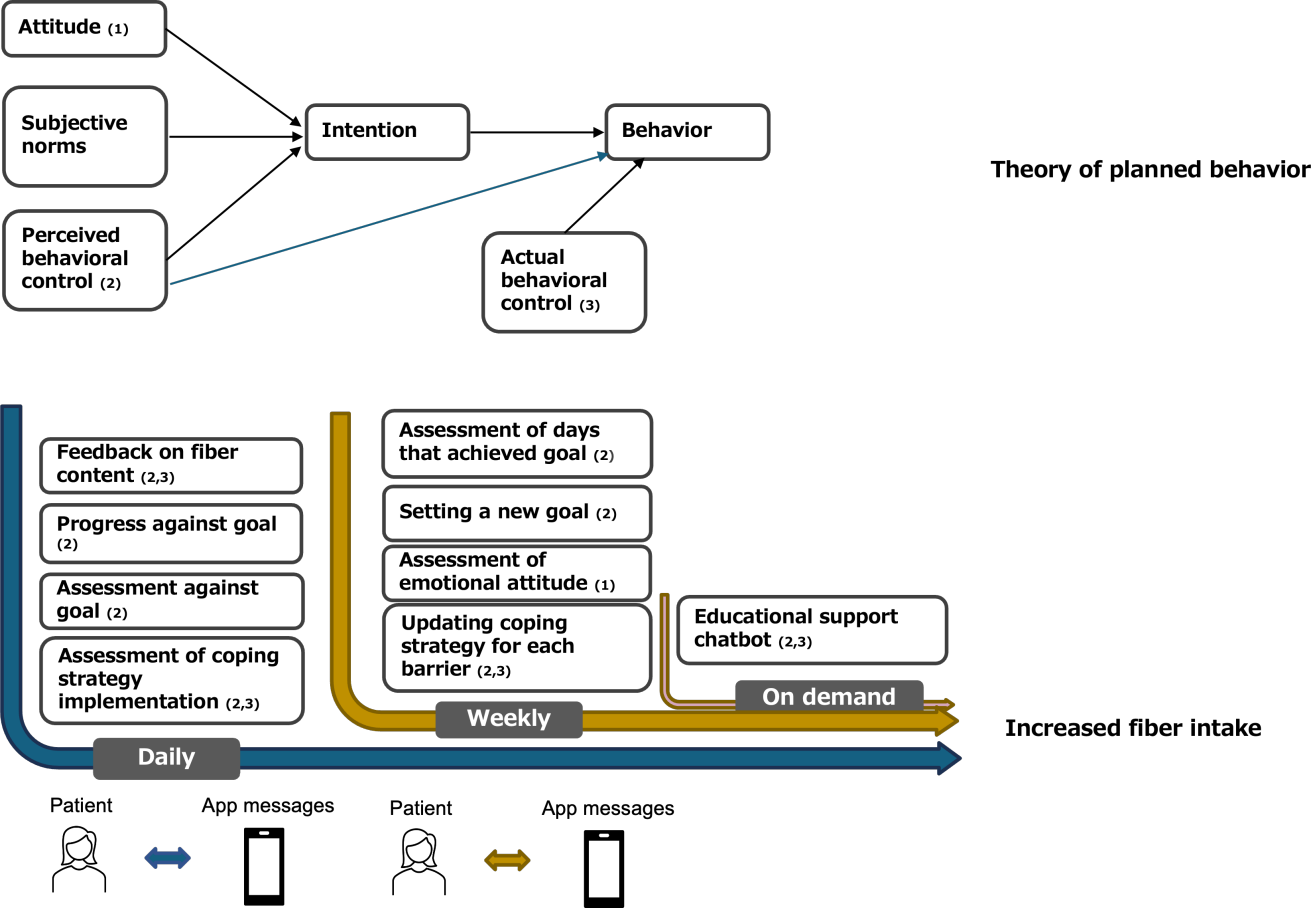
The theory of planned behavior (TPB) applied to the FiberMore intervention.

The FiberMore intervention is mainly designed to enhance PBC by targeting and modifying participants’ control beliefs, with the goal of influencing both intention and behavior (see Figure 3). This approach is informed by the findings from a formative study [35], which identified PBC as a significant predictor of participants’ intention to increase fiber intake. The formative research revealed that many participants lacked the skills needed to effectively implement this dietary behavior change, highlighting the need for tailored support to improve their sense of control and confidence.

The primary strategy involves the use of progressively challenging yet attainable goal-setting using a simple algorithm based on our previous work [20], which has been shown to enhance control beliefs and self-efficacy. Specifically, the chatbot suggests a new fiber intake goal each week, tailored to the participant’s performance in the previous week based on the number of days the goal was met (see Table 1).

Participants can monitor their daily progress toward their fiber goal through a real-time update graph displayed on the app’s home screen. Additionally, participants are able to view weekly goal achievement progress through this graph, supporting mastery experience and reinforcing a sense of achievement.

To further support PBC, participants receive personalized feedback on the fiber content and fiber-to-energy ratio of their meals. These feedback messages aim to increase participants’ awareness and perceived ability to make appropriate dietary choices, thereby reinforcing control beliefs. A third mechanism enhances PBC via daily self-assessment of coping strategy implementation and weekly setting of new barriers and corresponding coping strategies. Setting coping strategies can enhance the optimistic beliefs about one’s capability to cope with barriers that arise, leading to improved self-efficacy and a stronger sense of PBC when facing obstacles [43]. Another mechanism involves the “Learn More” chatbot, which provides useful information and tips on increasing fiber intake, aiming to enhance users’ confidence and perceived control over increasing their dietary fiber.

Beyond influencing PBC, the FiberMore intervention may also impact actual behavioral control (ABC). ABC measures the extent to which a person has the skills, resources, and other prerequisites needed to perform the behavior [44]. FiberMore’s provision of meal fiber values, daily fiber totals, and “Learn more” education on fiber may improve ABC by giving actionable information. To the extent that the solution offered to the barrier is new to the participant and effective, the barrier/solution process may increase ABC.

FiberMore also seeks to influence behavioral beliefs by shaping a positive emotional attitude toward the behavior, which in turn may increase users’ motivation and intention to perform the behavior [44]. This is implemented through a weekly “checking in” chatbot that encourages users to reflect on what aspects of increasing fiber intake have been enjoyable or challenging. In response to user input, the chatbot delivers empathetic messages, validates users’ experiences, and offers positive reinforcement to enhance emotional attitude toward increasing fiber intake.

We hypothesize that the FiberMore intervention will lead to improvements in participants’ attitude and PBC toward increasing dietary fiber intake. These changes are expected to enhance their intention to consume more fiber, which in turn will result in an increase in fiber intake (see Figure 3).

We will measure the impact of the FiberMore intervention on TPB outcomes via a questionnaire of 20 direct TPB belief measures of intention (questions 1‐5), attitude (questions 6‐11), subjective norms (questions 12‐14), and PBC (questions 15‐20) regarding eating more fiber-rich foods than usual. The scoring of each scale for the belief measures is 7-point from 1 to 7. This TPB questionnaire was developed with reference to a previously validated instrument targeting high-fiber dietary intake [45], and was guided by the TPB questionnaire development manual by Francis et al [46]. It has been previously tested among the T2D population in Japan and content-validated by diabetes specialists and researchers experienced in health behavior change research [35].

### Study Procedures and Participant Timeline

#### Preliminary Study Enrollment

The physicians at each research institution will conduct screening procedures to T2D patients who visited the outpatient clinic according to the inclusion and exclusion criteria of this study. A clinical research coordinator (CRC) will approach eligible patients to provide detailed information about this study and to obtain the patient’s written consent to participate in this study. Once consent to participate in this study is obtained, the CRC will assist study participants to download MealLog onto participants’ mobile phones and explain to study participants how to log meals through the app (see Table 2).

**Table 2.** Details of MealLog features.

Meal logging options in MealLog	Description
Snap a meal (or upload from phone gallery)	Study participants log a meal by taking a photo of the meal via the MealLog app. The meal photo is uploaded to the AI^a^ server and analyzed for its fiber, energy, carbohydrates, protein, fat, and salt amounts. No feedback on the meal is provided to the user via the app.
Chat a meal	Study participants describe a meal with the “Chat a meal” chatbot (EiYouBot) on the MealLog app to log the meal. Based on the description, the meal is analyzed by the AI server for its fiber, energy, carbohydrates, protein, fat, and salt amounts. No feedback on the meal is provided to the user via the app.
Snap a meal (or upload from phone gallery) followed by Chat a meal	Study participants first log a meal by taking a photo via the MealLog app. The meal photo is uploaded to the AI server and analyzed for its fiber, energy, carbohydrates, protein, fat, and salt amounts. No feedback on the meal is provided to the user via the app. Study participants then describe additional details about the meal with EiYouBot. Nutrient values are updated according to their added details.

a AI: artificial intelligence.

#### Run-in Period

During the run-in period, preliminarily enrolled study participants will use MealLog to log their meals so that the researchers can assess their average baseline fiber intake. A research co-investigator will contact the study participants via SMS text messages if participants do not log any meals or if only 1 meal is logged for 3 days consecutively since the start of the run-in period.

To be eligible for official study enrollment, study participants need to log at least 2 eating occasions (either main meals or snacks) in app per day for at least 7 days during the nominal 14-day run-in period and have an average baseline fiber intake of less than 20 g per day (after excluding days with 0 or 1 logged meal per day).

On the first 3 days of the run-in, at 11 AM, preliminarily enrolled study participants will receive an SMS containing a survey link for the TPB questionnaire (via Jotform). On day 1 of the run-in, study participants will receive a TPB questionnaire on increasing fiber intake. On day 2 of the run-in, they will receive a TPB questionnaire on reducing salt intake. On day 3 of the run-in, they will receive a TPB questionnaire on reducing saturated fat intake. Study participants are required to complete these surveys at home or at any convenient location with an internet connection. If the questionnaire submissions are not received by the 3rd day of the week, another SMS text message will be sent on the 4th day as a reminder to complete the missing questionnaire.

After completion of the run-in period, study participants will be contacted by the research office about their official study enrollment status.

#### Official Study Enrollment and Randomization

Randomization will be conducted using a dynamic allocation method based on minimization, with an allocation ratio of 1:1. The allocation adjustment factors will include the baseline dietary fiber intake during run-in (under 10 g, 10 g and above), sex (male and female), and HbA_1c_ level on the date of consent (less than 8.5% and 8.5% or higher). Random allocation will be performed online using the INDICE cloud system provided by the University Hospital Medical Information Network (UMIN). After randomization, a CRC will explain the details of the intervention according to the study participants’ allocated group (intervention or control). Participants are blind to their group.

#### The Intervention Condition

A CRC will assist study participants assigned to the intervention group with downloading FiberMore onto their smartphones and input the initial fiber goal of the study participants to the backend system (refer to Table 2 for determination of the initial goal). The CRC will also assist the study participants to identify 1 barrier on increasing fiber intake and its corresponding solution via the “Barrier and Solution” chatbot within FiberMore. The CRC will also provide guidance and demonstration on using the intervention features of FiberMore.

The CRC will give study participants a handout on the benefits of increasing fiber intake as well as general tips and examples about eating more fiber. As some study participants depend on their family for meal preparation, the CRC will encourage the study participant to share the educational handout with their family members and communicate the weekly fiber goal as well as the feedback obtained from FiberMore with their family or person preparing their meals.

On the first three days during week 10, at 11 AM, study participants will receive an SMS text message containing a survey link for a TPB questionnaire (via Jotform). On day 1 of week 10, study participants will receive a TPB questionnaire about increasing fiber intake. On day 2, they will receive a TPB questionnaire about reducing salt intake. On day 3, they will receive a TPB questionnaire about reducing saturated fat intake. Study participants are required to complete these surveys at home or any convenient location with an internet connection.

#### The Control Condition

**Figure 4.**
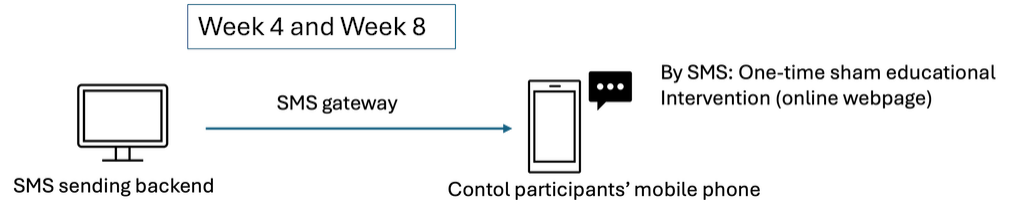
System and equipment used to send sham educational intervention to control participants by SMS text messaging (once during week 4 and once during week 8 of the intervention period).

**Figure 5.**
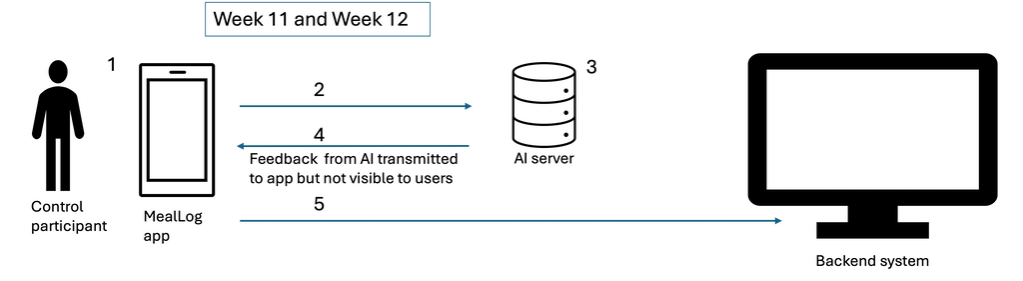
System and equipment used by the control participants every day during weeks 11 and 12 of the intervention period. AI: artificial intelligence.

After randomization, the control group will receive an educational handout on salt intake reduction as well as a paper diary to record their efforts in reducing salt intake. They are encouraged to record any small effort to reduce salt intake in the diary on a daily basis. They will also receive web-based educational information via SMS text message once during week 4 and once during week 8 (see Figure 4). On the last day of week 10, the control group will receive SMS text message reminders from the research office to use MealLog to log their meals for 14 days during week 11 and week 12 (see Figure 5).

On the first 3 days during week 10, at 11 AM, study participants will receive an SMS text message containing a survey link for a TPB questionnaire (via Jotform). On day 1 of week 10, study participants will receive a TPB questionnaire about increasing fiber intake. On day 2, they will receive a TPB questionnaire about reducing salt intake. On day 3, they will receive a TPB questionnaire about reducing saturated fat intake. Study participants are required to complete these surveys at home or any convenient location with an internet connection.

The flow of interaction between the intervention participant, the app interface, the backend system, and the AI server is as follows, as illustrated in Figure 5: the control participant logs meal via “Snap a meal” function or “Chat a meal” function in MealLog app during week 11 and week 12 of the intervention period (Step 1). Data provided by the participant is sent to the AI servers (Step 2). The AI servers process the uploaded meal photo or chatbot interaction and generate information on fiber, energy, carbohydrates, protein, fat, and salt amount (Step 3). Once the AI server completes processing, the app receives relevant feedback and data generated by AI. This feedback is not visible to the user within the app interface (Step 4). The mobile app then securely transmits the feedback to the backend system via internet connection using a highly encrypted API stored within the app (Step 5).

#### Study Events

This study includes 5 events (E0-E4), a 2-week run-in period, a 12-week intervention period, and a 12-week observation period. Study participant experience and data collected in different stages of the study are described in Table 3.

**Table 3.** Study events spanning approximately 26 weeks.

Event	Time	Key activities	
E0	Preliminary study enrollment	Participant experience: obtain consent to participate in study. Data collected: Patient background^a^, vital signs^b^, hematological tests^c^, biochemical tests^d^, urinalysis^e^, medication status survey, and the transtheoretical model (TTM) questionnaire for dietary behavior change^f^.	
Run-in period	From E0 for 2 weeks	Participant experience: Log meals with MealLog app Data collected: Number of days with at least 2 logged eating occasions (main meals or snacks), total fiber intake per day (grams), average fiber intake (excluding days with only 0 or 1 logged meal), theory of planned behavior (TPB) questionnaire for dietary fiber intake^g^, TPB questionnaire for salt intake^g^, and TPB questionnaire for saturated fat intake^g^.	
E1	At the time of official study enrollment	Intervention group: Participant experience: Download FiberMore and receive educational handout on dietary fiber intake. Data collected: Medication status and system defects.	
		Control group: Participant experience: Receive educational handout on salt reduction. Data collected: Medication status and system defects.	
Intervention period	From E1 for 12 weeks	Intervention group: Participant experience: Interact with FiberMore. Data collected: The number and percentage of days fiber goal is met out of 7 days, the number and percentage of implemented coping strategies per week, the average estimate of fiber intake per day (excluding days with only 0 or 1 logged meal), and the average estimate of energy intake per day (excluding days with only 0 or 1 logged meal).	
		Control group: Participant experience: Record salt reduction efforts in a paper diary (sham intervention), receive 1-time educational weblink on salt reduction via an SMS text message during week 4 and week 8, and use MealLog to log meals during week 11 and week 12. Data collected: During week 11 and week 12—the average estimate of fiber intake per day (excluding days with only 0 or 1 logged meal) and the average estimate of energy intake per day (excluding days with only 0 or 1 logged meal).	
E2	Week 10 of the intervention period	Participant experience: receive a survey link of TPB questionnaires about dietary fiber, salt, and saturated fat on specified day via SMS text messages.^g^ Data collected: TPB questionnaire for dietary fiber intake, TPB questionnaire for salt intake, and TPB questionnaire for saturated fat intake.	
E3	End of 12 weeks from E1 (end of the intervention period)	Participant experience: attend regular outpatient clinic appointment and attend E3 meeting with a clinical research coordinator (CRC). Data collected: vital signs, hematological examination, biochemical tests, urinalysis, medication status, system defect report, TTM questionnaire for dietary behavior change, and a survey on the participants’ satisfaction and perceived usefulness of the intervention.	
E4	End of 24 weeks from E1 (end of the observation period)	Participant experience: attend regular outpatient clinic appointment, attend E4 meeting with CRC, receive survey link of TPB questionnaires for dietary fiber, salt, and saturated fat on specified day via SMS text messages. Data collected: Vital signs, hematological examination, biochemical tests, urinalysis, medication status, and TTM questionnaire for dietary behavior change; TPB questionnaire for dietary fiber intake; TPB questionnaire for salt intake; TPB questionnaire for saturated fat intake; and a survey on the intention to improve eating habits using a generative artificial intelligence (AI)-enabled app.	

a Definitions related to observation, investigation, and reporting items: Patient background: (1) Via self-administered questionnaire: Highest education level, annual income, marital status, living arrangement, employment status, meal arrangement methods, control over meal ingredients and menu, level of daily physical activity; (2) Via medical record: Age, sex, date of diabetes diagnosis, smoking and alcohol consumption at the time of consent acquisition; the presence of macrovascular disease, diabetic retinopathy, periodontal disease, tinea pedis, and diabetic neuropathy; and other medical history.

b Vital signs: Body height, body weight, and systolic and diastolic blood pressure.

c Hematological tests (Performed at least a week before each time point): hemoglobin level.

d Biochemical tests (performed at least a week before each time point): estimate glomerular filtration rate (eGFR), creatinine, fasting blood glucose, HbA_1c_, low-density lipoprotein cholesterol (LDL-C), high-density lipoprotein cholesterol (HDL-C), triglycerides, albumin, blood urea nitrogen (BUN), and potassium.

e Urinalysis (performed at least a week before each time point): Urine protein and urine-albumin-creatine ratio (UACR).

f Transtheoretical model (TTM) questionnaire: Measure the 5 stages of dietary behavior change in TTM: Precontemplation, contemplation, preparation, action, and maintenance. Amount of dietary fiber intake: artificial intelligence (AI) assessment of dietary fiber intake via meals logged onto the app.

g The theory of planned behavior (TPB) questionnaire: Measure attitude, subjective norms, perceived behavioral control, and intention to improve dietary behavior.

### Study Outcomes

#### Primary Outcome

The primary outcome of this study is the between-group difference in the change in daily intake of dietary fiber from the time of run-in to the end of the intervention period.

#### Secondary Outcomes

Secondary outcomes (see Table 4) include clinical and health measures, measurements of behavior changes, engagement measures with intervention features, and assessments of participants’ satisfaction and perceived usefulness of the intervention. We will also assess the occurrence of adverse events via patient interviews.

**Table 4.** Secondary outcome measures.

Measurements	Outcomes
Difference in changes	HbA_1c_ (%)^a^ Weight (kg)^a^ BMI (kg/m^2^)^a^ Systolic and diastolic blood pressure (mmHg)^a^ Estimate glomerular filtration rate (eGFR; ml/min/1.73m2)^a^ Fasting blood glucose (mg/dL)^a^ Low-density lipoprotein cholesterol (LDL-C; mg/dL)^a^ High-density lipoprotein cholesterol (HDL-C; mg/dL)^a^ Triglyceride (mg/dL)^a^ Theory of planned behavior (TPB) elements for dietary fiber, salt, and saturated fat intake (attitude, subjective norm, perceived behavioral control, and intention).^b^
Difference in proportions	Transtheoretical model (TTM) stage of change questionnaire for dietary behavior.^a^ Changes in antidiabetic drugs since consent was obtained (intensified, unchanged, and weakened).^a^ Usage of each antidiabetic drug.^c^
Measured values	Average estimate of fiber intake per day.^d^ Average estimate of energy intake per day.^d^ Number and percentage of days fiber goal is met out of 7 days.^d^ Number and percentage of implemented solutions in a week.^d^ Survey on participants’ satisfaction and perceived usefulness of the intervention.^e^ Percentage of patients with HbA_1c_ less than 7%.^f^ Survey on intention to improve eating habits using a generative artificial intelligence (AI)-enabled app^g^

a Difference in change from the time consent was obtained to the end of the intervention period (12 weeks) and to the end of the observation period (24 weeks).

b Difference in change from the time of run-in to the end of 10 weeks and to the end of the observation period (24 weeks).

c Calculated at the time of consent acquisition, at the time of official study enrollment, after the end of the intervention period, and after the end of the observation period.

d Evaluated weekly from week 1 to 12 for the intervention group.

e Percentages of responses for each option on the Likert scale and for the Yes or No scale will be calculated after the end of the intervention period for the intervention group and control group.

f Evaluated at the end of week 12 and week 24 for both intervention group and control group.

g Percentages of responses for each option on the Likert scale will be calculated after the end of the observation period for the intervention group.

### Sample Size Consideration

The primary outcome is the difference between the intervention group and the control group in the change in mean daily dietary fiber intake from the time of study enrollment to the end of the intervention period. We desired to detect a between-group difference in the mean change of 7 g of dietary fiber, drawing from a meta-analysis, which demonstrated a 9% risk reduction of cardiovascular disease events for 7 g increase in daily dietary fiber intake [47]. A previous RCT reported a 7.9 g/day increase in fiber intake at 3 months in the high-fiber group, supporting the use of a 7 g/day between-group difference as a realistic and meaningful target [33].

Based on the findings from a previous nutritional intervention research in Japan [48], we assumed a common SD of 9.85 g for the difference in dietary fiber change across both groups. Using a 2-sided significance level of 5% and a power of 80%, the required sample size calculated based on an independent *t* test is 33 participants per group. A study conducted in a similar population, which followed a similar study structure with a 2-week run-in phase, a 12-week intervention phase, and a subsequent 12-week observation phase by our research team showed minimal to no dropout throughout the study duration [49]. Nevertheless, as a conservative estimate to account for potential attrition, we aimed to enroll a minimum of 70 participants. The 2-week run-in phase also ensures that we enroll the participants who are able to use the app as instructed, thus minimizing drop-out risk.

Based on the findings from previous studies implemented in our research lab, we estimate a 10% dropout rate between preliminary and official study enrollment. Therefore, the target recruitment number for the preliminary study enrollment has been set at 77 participants.

### Statistical Analysis Plan

In this study, we define 3 analysis populations: the full analysis set (FAS), the per protocol set (PPS), and the safety analysis set (SAS). The FAS is defined by all the study participants who had at least one primary outcome or secondary outcome obtained after randomization. We will follow the intention-to-treat (ITT) principle and analyze data based on the assigned group in the FAS analysis. The PPS is the FAS population with exclusion of patients who were found to have serious violations of the provisions of the research protocol:

1. Violation of inclusion and exclusion criteria
2. Study participants who log fewer than 2 meals per day on more than 7 days within any of the following 2-week intervals during the intervention period: Weeks 1‐2, 3‐4, 5‐6, 7‐8, 9‐10, 11‐12, or 13‐14.
3. In the event of a medical condition that makes dietary fiber intake inappropriate for the study participants,

Major deviations from other provisions of the study protocol will be assessed through a blinded review process. The blinded review will be conducted according to the following procedures: assessment of whether individual participants or specific measurement data should be excluded from the PPS or FAS; evaluation of participants with significant protocol deviations to determine whether exclusion is warranted; and examination of efficacy outcomes, including consideration of potential variable transformations and identification of outliers.

The safety analysis set is the set of all patients who used either MealLog or FiberMore at least once after randomization. In the safety analysis using the SAS, we will analyze data based on the intervention actually used by patients regardless of allocation. The FAS will be the primary analysis population, and the PPS will provide supportive results. All safety analyses will be conducted in the SAS. Data on patients’ characteristics will be presented as mean, minimum, SD, 25th percentile, median, 75th percentile, and maximum for continuous variables and as frequency and proportion for categorical variables.

### Primary Outcome Analysis

We will perform analysis of covariance on the change in dietary fiber intake from the time of run-in to the end of the intervention period, with dietary fiber intake from the time of run-in as the covariate. The change in dietary fiber from the time of run-in and at the end of the intervention period will be calculated as ([average dietary fiber over week 11 and 12]-[average baseline dietary fiber during the run-in]). The estimated mean change in each group and its 2-sided 95% CI will be calculated. Additionally, the estimated difference between groups and its 2-sided 95% CI will be calculated.

To ensure reliable estimates of fiber intake, days with only 0 or 1 logged meal will be excluded from the analysis.

### Secondary Outcomes Analysis

We will perform analysis of covariance on the change of HbA_1c_ from the time consent is obtained to the end of the intervention period (at 12 weeks) or the end of the observation period (at 24 weeks), using the measurements at the time consent is obtained as covariates. The estimated mean change in each group and its 2-sided 95% CI will be calculated. Additionally, the estimated difference between groups and its 2-sided 95% CI will be calculated. The proportion of HbA_1c_ less than 7% at week 12 (E3) and week 24 (E4; or point of discontinuation, if earlier) will be compared between groups by the Fisher exact test.

The change from baseline in the clinical laboratory test values stated below at week 12 (E3) and week 24 (E4) (or point of discontinuation, if earlier) will be analyzed as the secondary outcomes:

1.  Weight (kg)
2.  BMI (kg/m^2^)
3.  Systolic and diastolic blood pressure (mmHg)
4.  Estimated glomerular filtration rate (ml/min/1.73 m^2^)
5.  Fasting blood glucose (mg/dL)
6.  Low-density lipoproteincholesterol (LDL-C; mg/dL)
7.  High-density lipoproteincholesterol (HDL-C; mg/dL)
8.  Triglyceride (mg/dL)

The percentages of participants at each stage of the transtheoretical model (TTM) stage of change will be calculated for both groups at the end of the intervention period (week 12) and the end of the observation period (week 24) were compared between groups using the Cochran-Mantel-Haenszel test.

Between-group changes in all TPB survey elements for dietary fiber, salt, and saturated fat intake (attitude, subjective norms, PBC, and intention) will be analyzed from the time of run-in to the end of 10 weeks, and to the end of the observation period (24 weeks), using repeated measures ANOVA with time (Week 1, Week 10, and Week 24) as a within-subjects factor and group (control vs intervention) as a between-subjects factor.

Changes in diabetes medications since consent acquisition at the end of the intervention period (at 12 weeks) and at the end of the observation period (at 24 weeks; classified as intensified, unchanged, and weakened) will be calculated for each group and compared between groups using the Cochran-Mantel-Haenszel test. The proportion of new medications added will be compared between groups by the Fisher exact test.

We will also calculate the percentage of responses for each option on the Likert scale and for the “Yes or No” scale in “Survey on participants’ satisfaction and perceived usefulness of the intervention” at the end of the intervention period for both intervention group and control group. For the “Survey on intention to improve eating habits using a Generative AI-enabled app”, we will calculate the percentage of responses for each option on the Likert scale at the end of the observation period for the intervention group.

As part of our exploratory analysis, we will assess the reliability of the TPB questionnaire using the Cronbach α coefficient. Additionally, we will conduct multivariate regression analyses to examine various associations related to the TPB constructs. Specifically, we will investigate the relationship between changes in intention (INT) to increase fiber intake and changes in fiber intake outcomes both between and within groups. Furthermore, we will analyze the associations between changes in attitude (ATT), subjective norms (SN), and PBC with changes in intention to increase fiber intake, as well as the relationship between changes in PBC and changes in fiber intake outcomes at both the between-group and within-group levels. The impact of the intervention on INT via ATT, SN, and PBC will be evaluated through mediation analysis.

We will calculate the frequency and weekly percentages of the following measurements during the intervention period in the intervention: number and percentage of days fiber goal is met out of 7 days, the number and percentage of implemented solutions in a week, the average weekly fiber change in the intervention group from run-in, and the average weekly energy change in the intervention group from run-in. We will also evaluate the effects of changes in dietary fiber intake on changes in BMI and HbA_1c_ using the Pearson correlation test and simple regression analysis.

We will perform a statistical analysis of safety outcomes by calculating the number and percentage of individuals who experience adverse events during run-in, intervention, and observation periods. During the intervention and observation periods, data will be compiled separately for the intervention and control group, and the percentages will be compared between groups using the Fisher exact test.

We will not apply statistical adjustment for multiple comparisons in the analysis of secondary and exploratory outcomes. We will interpret these results as exploratory and use them to inform future hypotheses.

### Monitoring, Quality Control, and Data Management

An independent auditor, separate from the departments involved in this clinical trial, will inspect the medical institution and other relevant facilities to ensure that the trial is conducted in compliance with established protocols and standards.

In accordance with the EU Artificial Intelligence Act [50], FiberMore falls under the limited-risk category, as it delivers dietary advice and behavioral support but does not make clinical diagnoses or treatment decisions. We implemented a few measures to ensure that the dietary advice and behavioral support offered in FiberMore is safe for T2D patients. FiberMore has undergone pretesting and has been reviewed by a diabetes specialist on the research team to ensure its suitability for providing dietary advice to individuals with diabetes. The intervention development team has implemented safeguards to ensure that the chatbot’s conversations are suitable and reliable. For example, in the Learn More chatbot where users can get educational information, the chatbot discloses that “As I am a bot, I may rarely make mistakes, so please always use your common sense and follow your doctor’s instructions.” In addition, the chatbot conversations will be monitored by the study team daily to ensure that the advice given is safe and appropriate to T2D patients. If study participants encounter any unusual response from the chatbot at any time, they are advised to take a screenshot of the issue and send it to us by email, SMS text message, or Line, a popular social messaging app in Japan.

All data measured and provided through FiberMore will be securely transmitted to the research office’s backend system, which is hosted on Firebase, a cloud-based platform developed by Google on a password-protected computer through an internet connection and stored on the backend system. Firebase complies with major regulatory frameworks such as the General Data Protection Regulation and the California Consumer Privacy Act, meets international security and privacy standards including ISO/IEC 27001 (information security management) and ISO/IEC 27018 (protection of personally identifiable information in the cloud), and aligns with requirements under Japan’s Act on the Protection of Personal Information [51,52].

No personally identifiable information will be stored in the backend system alongside the study data, and all information will be pseudonymized using study participant ID. Data stored in the backend system include study participants’ ID, meal photos taken by study participants and photo timestamp, chat logs generated in FiberMore and its timestamp, feedback on nutritional information generated by AI on each logged meals, total daily intake and average weekly intake of energy, dietary fiber, and other nutrients (eg, protein, fat, carbohydrates, salt), daily fiber goal achievement status, daily implementation status of coping strategies (solutions), and content of weekly chosen barrier and coping strategies to increase fiber intake. All data on the backend system will be automatically backed up once a day across Google Cloud’s secure data centers. These backup copies can be restored in the event of system failure, accidental deletion, or data corruption.

Survey data from paper questionnaires are entered into the study database by a research co-investigator and independently verified by another research co-investigator to ensure data accuracy. Paper files are stored in locked cabinets within a locked room and are accessible only to authorized personnel. Paper questionnaires are stored in a locked room in locked cabinets and can be accessed only by authorized personnel.

TPB questionnaires administered using Jotform will be transmitted to the Jotform server, where the data is stored and accumulated. Jotform supports Health Insurance Portability and Accountability Act (HIPAA) compliance for managing sensitive health data, complies with GDPR for handling personal information, and aligns with the principles of Japan’s Act on the Protection of Personal Information [53]. Surveys administered via Jotform are pseudonymized using study participant ID. A research co-investigator will download the survey responses from the Jotform server into a password-protected folder on a computer in the Department of Planning, Information, and Management of The University of Tokyo Hospital for data analysis. To maintain data quality, participants with missing responses or inconsistencies on the TPB questionnaire such as extreme or contradictory answers to items assessing the same underlying construct will be asked to recomplete the relevant survey items.

### Ethical Considerations

This trial will be conducted in compliance with the Declaration of Helsinki, the Pharmaceutical and Medical Device Act, the Ministerial Ordinance on Good Clinical Practice for Medical Devices, and all other guidelines in relation to these regulations (jRCT2032240579). This study was approved by the institutional review board of the University of Tokyo School of Medicine (approval 2024442NI).

Before the start of the study, we provide clear explanations to the study participants using the consent explanation document. Written consent, based on the study participant’s free will, will be obtained. When obtaining consent, study participants will be given sufficient time and opportunities to ask questions, and all questions will be answered thoroughly. We also inform the study participants that their meal logs and chat conversations with the bot will be monitored by the research team for quality assurance and to improve the future iterations of the system. All patient data will be pseudonymized.

Study participants are free to withdraw consent at any point of the study, either pre- or postrandomization by contacting the research office in writing or via phone. We also inform the study participants that if consent is withdrawn before randomization, all the collected data will be discarded, while if consent is withdrawn after randomization, data collected up to the point of withdrawal will be retained.

As compensation for their participation, transportation costs to the research sites, phone internet data cost, and the time and effort required to complete the study procedures, study participants will receive a QUO card worth 30,000 yen (approximately US $200) after completing the 24-week observation period and the related questionnaires. This amount is considered reasonable for a 6-month clinical study in Japan. It is not intended as an inducement to participate but rather as a token of appreciation and to reduce financial barriers to participation.

All blood test and health examination results will be reviewed by the participant’s primary physician (also the recruiting physician) during routine visits, with results communicated to the participant during their outpatient consultation. If a study participant reports any concerning symptoms to the CRC, the CRC will consult the principal investigator, who is a diabetes specialist. If deemed necessary, the participant will be referred to their primary physician for further evaluation or care.

To ensure equal educational opportunities for all participants without influencing study outcomes, participants in the intervention group will receive a handout on salt reduction strategies at the postobservation (E4) meeting, while those in the control group will receive, also at E4, a handout detailing the benefits of increased dietary fiber intake, general dietary tips, and examples of high-fiber foods.

## Results

Recruitment began on February 12, 2025, and ended on September 1, 2025. We anticipate the intervention period will conclude in December 2025 and the observation period will conclude in March 2026.

**Figure 6.**
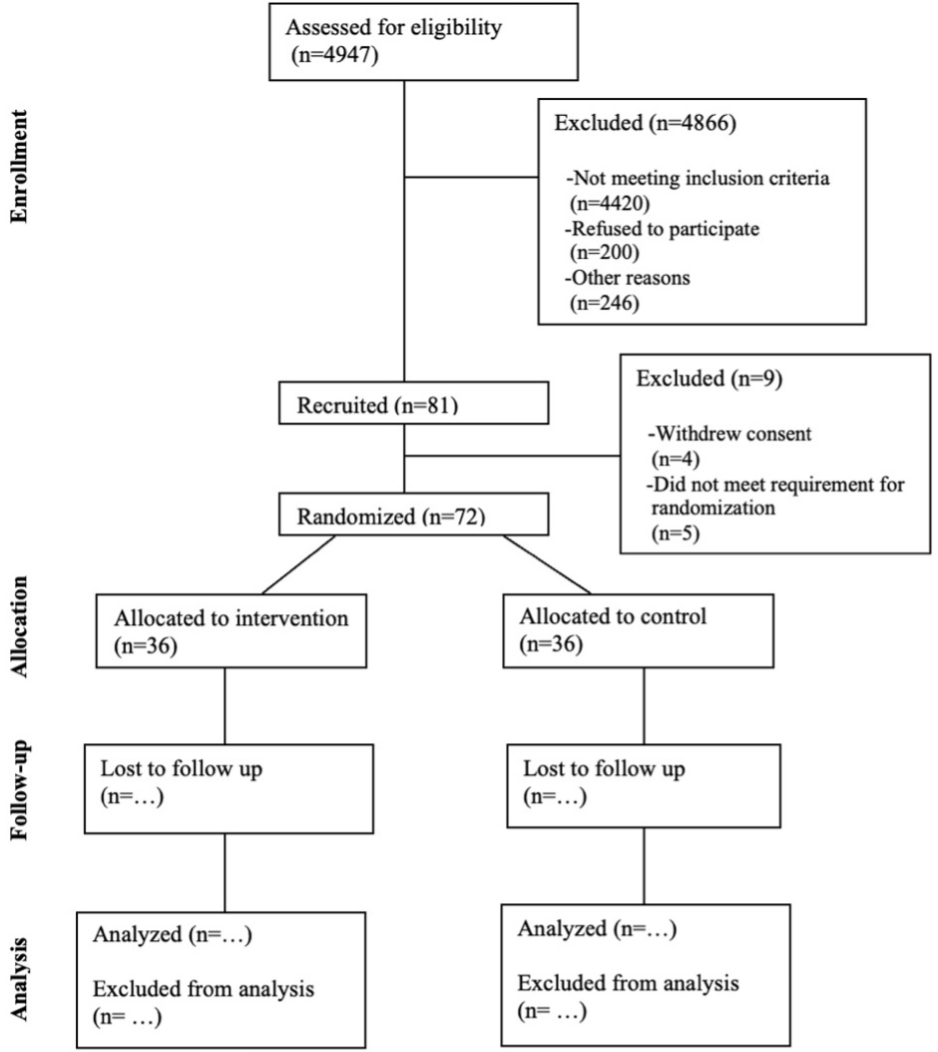
CONSORT flow diagram depicting enrollment, allocation, follow-up, and analysis phases as of September 22, 2025.

As of September 22, 2025, 4947 patients have been screened for eligibility and 81 participants have been recruited to the study. A total of 72 participants have been officially enrolled and randomized. Progress through the phases of this planned randomized trial (enrollment, intervention allocation, follow-up, and data analysis) will be visually represented using the CONSORT (Consolidated Standards of Reporting Trials) flow diagram (see Figure 6).

## Discussion

This study proposes an mHealth intervention aiming to assist T2D patients in increasing their dietary fiber intake and possibly improving their glycemic control. In the management of diabetes, achieving glycemic control is critical for the prevention of complications, with a treatment goal of maintaining HbA_1c_ levels below 7% [15]. Despite advances in pharmacotherapy aimed at improving glycemic control, the increasing aging population with diabetes has raised concerns regarding the potential adverse effects and risks of polypharmacy associated with prolonged medication use [54]. The Diabetes Treatment Guide also specifies the need to continue lifestyle self-management such as diet and exercise regimens even when drug therapy is initiated [15]. This intervention could potentially enhance nutritional awareness and promote healthy eating habits among T2D patients.

The AI-assisted dietary fiber intervention was developed using a user-centered design approach, informed by recent formative research conducted before this study [35]. The formative research highlighted the usefulness of TPB in predicting intention to increase dietary fiber intake among individuals with T2D, with PBC and attitude emerging as significant predictors of this intention. These insights guided the design and refinement of the FiberMore intervention to better align with the needs and motivations of the target population. Previous meta-analyses have also demonstrated the capacity of the TPB to predict dietary behaviors [55,56]. We anticipate that the FiberMore intervention will increase PBC and attitude of patients toward fiber intake, which in turn will increase their intention and subsequently lead to increased fiber consumption.

The FiberMore intervention incorporates a personalized goal-setting approach, in which the user’s fiber goal increases gradually based on the number of days in the current week that the goal is successfully achieved. This approach is modeled after the success demonstrated in a previous study that used this goal-setting strategy, where incremental increases in step count goals were achieved and accompanied by corresponding growth in actual step count throughout the intervention period [20]. Additionally, during the first week of the intervention, intervention group participants will be given an initial fiber goal based on the 10th highest daily fiber intake achieved during the run-in period, to ensure that the initial fiber goal is achievable. As repeated failure to meet a goal can result in diminished confidence over goal achievement [57], the intervention begins with an easily attainable goal to foster a sense of success and strengthen participants’ perceived control over goal attainment. The goal-setting algorithm will then recommend gradual goal increments once participants successfully meet their fiber goals for at least 5 days in a week.

FiberMore allows users to add on additional information on the meal photos, such as the portion size of the food and details not visible in meal photos to enhance the accuracy of the nutrient estimation. Given that forgetting to photograph meals is a commonly reported issue in digital food logging using photography [58], the “chat a meal” feature provides an alternative way for participants to log their meals, thus reducing chances of missing meal records.

FiberMore is designed with patient safety in mind by minimizing gastrointestinal effects that may arise with increased dietary fiber intake. The intervention motivates study participants to increase their fiber in a gradual manner, rather than requiring a large amount of fiber intake all at once. This gradual approach allows the digestive system to adapt slowly to the increased fiber, thereby reducing the likelihood of gastrointestinal discomfort while allowing time to adjust to new eating habits. We have used prompt engineering to ensure that the maximum goal increase from the previous week’s goal is 2 g and cap the maximum fiber goal that can be set to 30 g per day.

One of the key features of this study is the use of salt reduction educational intervention as a “sham intervention” for the control group. Salt reduction education is a widely accepted strategy in the management of T2D [41]. It is anticipated that participation in a study will introduce biases that influence participant behavior, a phenomenon known as the “Hawthorne effect.” Intervention participants may be more conscious of their behavior if they believe that they are receiving a more beneficial intervention, while control participants may be less likely to comply with the trial protocol if they believe that they are not receiving active treatment [59]. Through the implementation of a sham intervention, the Hawthorne effect of receiving an intervention can be controlled [42]. We do not anticipate that the salt reduction educational intervention will have a significant impact on fiber consumption, as the educational content specifically focused on reducing sodium intake through cooking methods and seasoning practices, without targeting food choices related to dietary fiber.

To maintain blinding, participants will be informed that the study aims to assess the usefulness of a smartphone app in improving the eating habits of patients with type 2 diabetes. They will not be told that the intervention specifically targets increasing dietary fiber intake. Additionally, participants will remain unaware that they are randomly assigned to one of two groups. This approach helps mitigate social desirability bias, allowing for a more objective assessment of the intervention’s effects and enhancing the overall validity of the study’s findings.

Another key aspect of our approach is focusing exclusively on patients who are already motivated to change their behavior in dietary fiber intake. We achieve this by filtering participants based on their stage of change in TTM, targeting those in the contemplation, preparation, and action stages, which has shown to be effective [20,60]. Individuals who are already motivated to change need guidance and support to enhance their intentions and to translate their intentions into action, and they are more likely to engage with self-management tools [61]. In contrast, individuals with low motivation would require a fundamentally different intervention focused on enhancing motivation. Our strategy is supported by a study that reported 93.7% of T2D patients in Japan fall within these stages of dietary behavior change [62], suggesting that our intervention approach applies to a very large group of potential participants.

The study will maintain usual care practices for both the intervention and control groups. There will be no restrictions on medication adjustments or concurrent treatments during the study period. The intervention is aimed exclusively at improving dietary fiber intake, complementing the dietary counseling that T2D patients typically receive as a standard care practice in Japan.

This study is a multicenter randomized controlled trial, which enhances the generalizability of the findings to individuals with T2D across Japan. The elements of the intervention, such as the use of an AI-enabled chatbot to carry out personalized goal setting, barrier-solution planning, and meal logging are not unique to the Japanese context and can be adapted to other settings.

This study has several limitations. First, the findings may be specific to the studied population and may not fully generalize to other populations. Since the participants are likely to be older individuals of Japanese ethnicity, differences in lifestyles and the pathophysiology of T2D between Japanese and other populations may limit broader applicability. The cultural and dietary context of Japan may limit generalizability of the specific dietary patterns observed outside Japan. Additionally, the study is restricted to participants who can use smartphones; therefore, individuals with lower technological proficiency may be underrepresented. Individuals from lower socioeconomic backgrounds may have limited access to smartphones or may rely on older devices with limited functionality, which may result in selection bias that underrepresents these individuals.

AI performance in meal assessment can be significantly influenced by image quality, lighting conditions, and the angle at which the photo is taken [37]. Since participants use their own smartphones to capture meal images, variability in phone models and camera quality may introduce inconsistencies in photo quality. However, this approach closely mimics real-world conditions, making the findings more applicable to practical settings. To mitigate this variability, the study team provides detailed guidance and education to participants on how to take clear and well-angled photos to optimize AI performance.

Blinding of researchers is not considered in this study. Due to the complex nature of a dietary behavior change intervention that necessitates interactions between the researcher and participants, implementing researcher blinding is not feasible in this study, which may impose some degree of bias in the intervention delivery. The intervention content feedback and questionnaire reminders are delivered primarily by the app through prespecified algorithms and automated messages, thus minimizing researcher involvement in the intervention delivery. Furthermore, separate teams handle participant support and data management, and the statistician will remain blinded throughout the study.

The 24-week study involves multiple study events and intervention procedures, which may increase the risk of participant fatigue and dropout. We mitigate this by providing thorough explanation during study enrollment, along with a take-home manual containing detailed instructions on study procedures. To support adherence, we use SMS reminders and the LINE messaging app to notify participants of their hospital visits and intervention schedules. In addition, we align all study visits with routine hospital appointments, except for the single visit at official enrollment, thereby minimizing participant burden.

In this study, we will recruit T2D patients with an HbA_1c_ of at least 7.5%, thereby limiting the generalizability of the study findings to the broader T2D population. Nevertheless, as the treatment target for HbA1c is generally <7% according to the JDS guideline, this cut-off allows us to target patients who are most likely to benefit from additional dietary support. We also exclude patients with chronic kidney disease stage 4 and above to minimize the risk of potential hyperkalemia associated with fiber-rich foods, which may exclude a substantial portion of T2D patients.

Manual daily monitoring of the chatbot responses will be performed by the study team as a quality assurance measure, and to comply with ethics committee requirements. We acknowledge that this process may not be scalable in future larger-scale implementations. To address this limitation, future iterations of the system will explore the integration of automated monitoring features to reduce the burden on human resources.

The AI-based meal image analysis used to estimate dietary fiber and energy intake is not a validated gold-standard method, such as weighed food records, which may introduce bias in the dietary estimates. Nevertheless, our estimation approach is grounded in previous validation work comparing the AI model’s outputs to dietitian assessments, which demonstrated acceptable accuracy for fiber and energy estimation [39]. Furthermore, the same assessment method is applied uniformly to both the intervention and control group, so that any potential measurement error introduced by the AI model is unlikely to cause a bias to between-group comparisons.

We acknowledge that AI may misclassify food items and also portion sizes, which may introduce bias in the dietary estimates. These image recognition models are typically trained on datasets featuring popular or standardized foods, which may limit their ability to accurately classify ingredients in less common meals, such as regional specialties, traditional home-cooked dishes, or foods from rural areas. Nevertheless, the AI model used in this study has been fine-tuned using a dataset of real-world meal photos, resulting in improved accuracy in estimating dietary fiber content. Our fine-tuned AI model demonstrates better accuracy in estimating fiber and energy content from meal photos than dietitians, whose estimation capabilities are widely regarded as an acceptable standard for validating dietary intake [55]. Since our AI model exceeds expert-level accuracy in estimating dietary fiber and energy content from meal images, we believe that the model is sufficiently reliable for the purposes of this intervention.

This pilot RCT will provide important evidence on the efficacy, feasibility, and safety of an AI-based mHealth intervention in improving dietary fiber intake and glycemic control among free-living T2D patients. This pilot study offers valuable insights and evidence on the development of AI as a promising method for accurate, low-burden dietary assessment [37], including how participants engage with AI-based meal logging and respond to AI-driven diet intervention. Additionally, this study has the potential to generate a large volume of meal photos taken in real-world conditions, which can be leveraged for further fine-tuning and improving the AI model’s accuracy and performance in analyzing user-generated meal images.

## Supplementary material

10.2196/78019 Multimedia Appendix 1 Calculation of target energy intake in FiberMore.

10.2196/78019 Multimedia Appendix 2 Overview of participant interaction with FiberMore.

## Abbreviation

ABC: actual behavioral control
AI: artificial intelligence
CRC: clinical research coordinator
FAS: full analysis set
JDS: Japan Diabetes Society
PBC: perceived behavioral control
PPS: per protocol set
T2D: type 2 diabetes
TPB: theory of planned behavior
