# Supplementary material for: Efficacy of FiberMore, an AI-Based mHealth Intervention to Increase Dietary Fiber Intake Among Type 2 Diabetes Patients: Protocol for a Pilot Randomized Controlled Trial
Source: JMIR Res Protoc. 2025 Dec 4;14:e78019. doi: 10.2196/78019 (PMC12677880; doi:10.2196/78019)
Supplement: Multimedia Appendix 1 [file resprot-v14-e78019-s001.docx]

**Appendix II**

**Calculation of target energy intake in FiberMore**

Target energy intake is calculated according to the formula recommended by JDS [Araki, 2020][18]:

Target energy intake(kcal/day)= Target body weight** (kg)× Energy coefficient(kcal/kg) according to level of physical activity

- ① Light exertion (static activity engaged mostly in the seated position): 25–30***
- ② Ordinary exertion (mainly static activity engaged in the seated position including commuting, household chores, and light exercise): 30–35***
- ③ Heavy exertion (heavy physical work or habitual, active exercise): 35~

** Target body weight is determined by the formula below:

Height(m)^2^×22

***The lower end of the range of energy coefficient is used in the calculation of target energy intake in this study.
