# Supplementary material for: Efficacy of FiberMore, an AI-Based mHealth Intervention to Increase Dietary Fiber Intake Among Type 2 Diabetes Patients: Protocol for a Pilot Randomized Controlled Trial
Source: JMIR Res Protoc. 2025 Dec 4;14:e78019. doi: 10.2196/78019 (PMC12677880; doi:10.2196/78019)
Supplement: Multimedia Appendix 2 [file resprot-v14-e78019-s002.docx]

**Appendix 1: Overview of Participant Interaction with FiberMore**

**Step 1: Daily feedback on previous day’s fiber goal achievement status and assessment of implementation status of coping strategies**

Upon opening the app on a new day, study participants will receive feedback on a pop-up screen regarding whether they met their dietary fiber goal on the previous day.

On the same pop-up screen, participants will be presented with the current selected barrier to increasing fiber and the associated solution (coping strategy) that they have selected for that week, with a message: "Did you try your chosen solution yesterday?" (Screenshot 1a,1b). Study participants are required to select either Yes or No. Upon selection, the chatbot will generate encouraging messages on the Home screen of the app (Screenshot 2a, 2b).


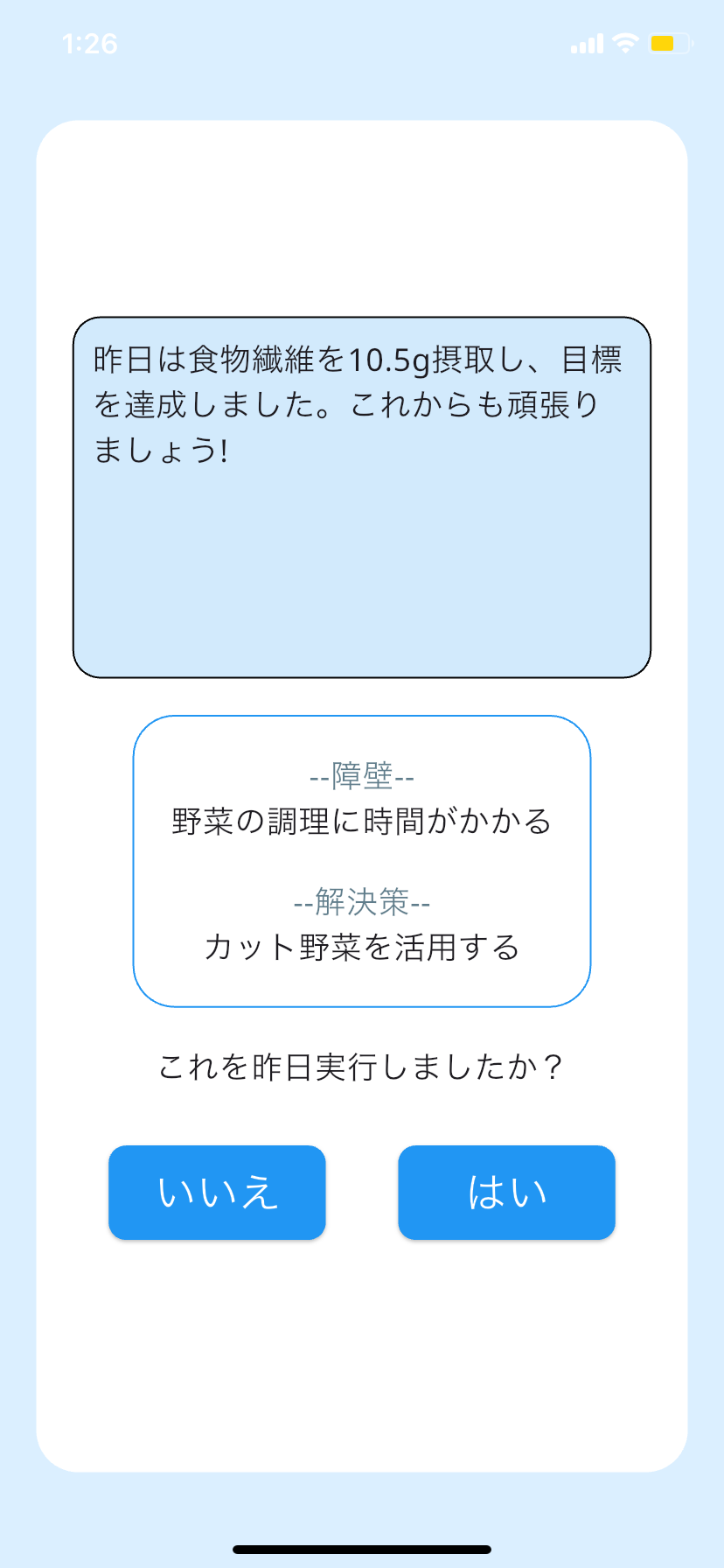


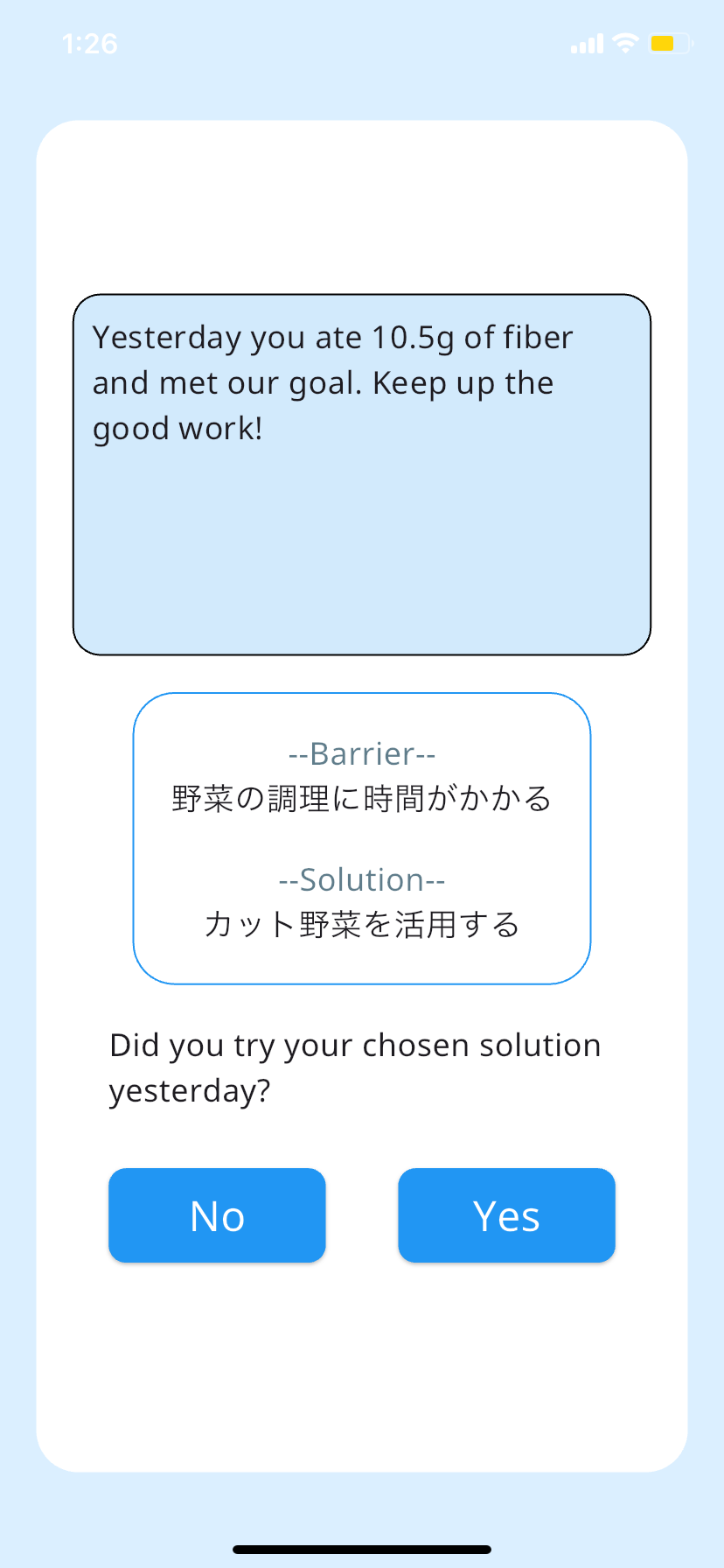


Cooking vegetables takes time

Use pre-cut vegetables

Screenshot 1a: Daily feedback on previous day’s fiber goal achievement status and assessment of implementation status of solution (Japanese)

Screenshot 1b: Daily feedback on previous day’s fiber goal achievement status and assessment of implementation status of solution (English)


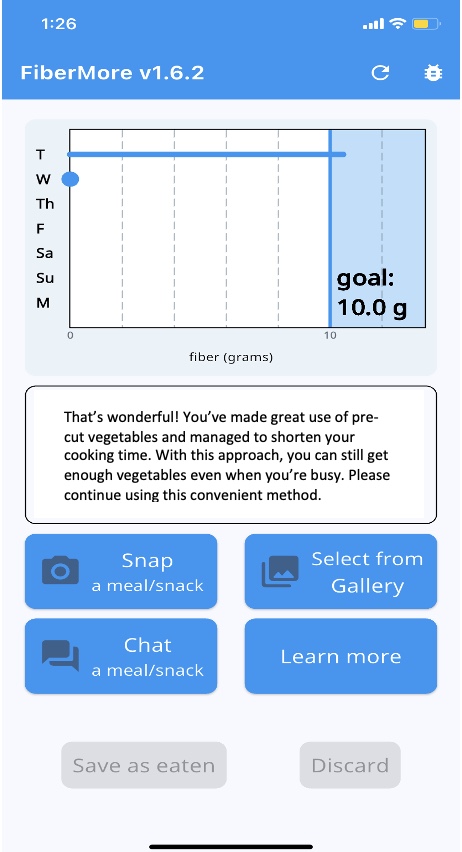


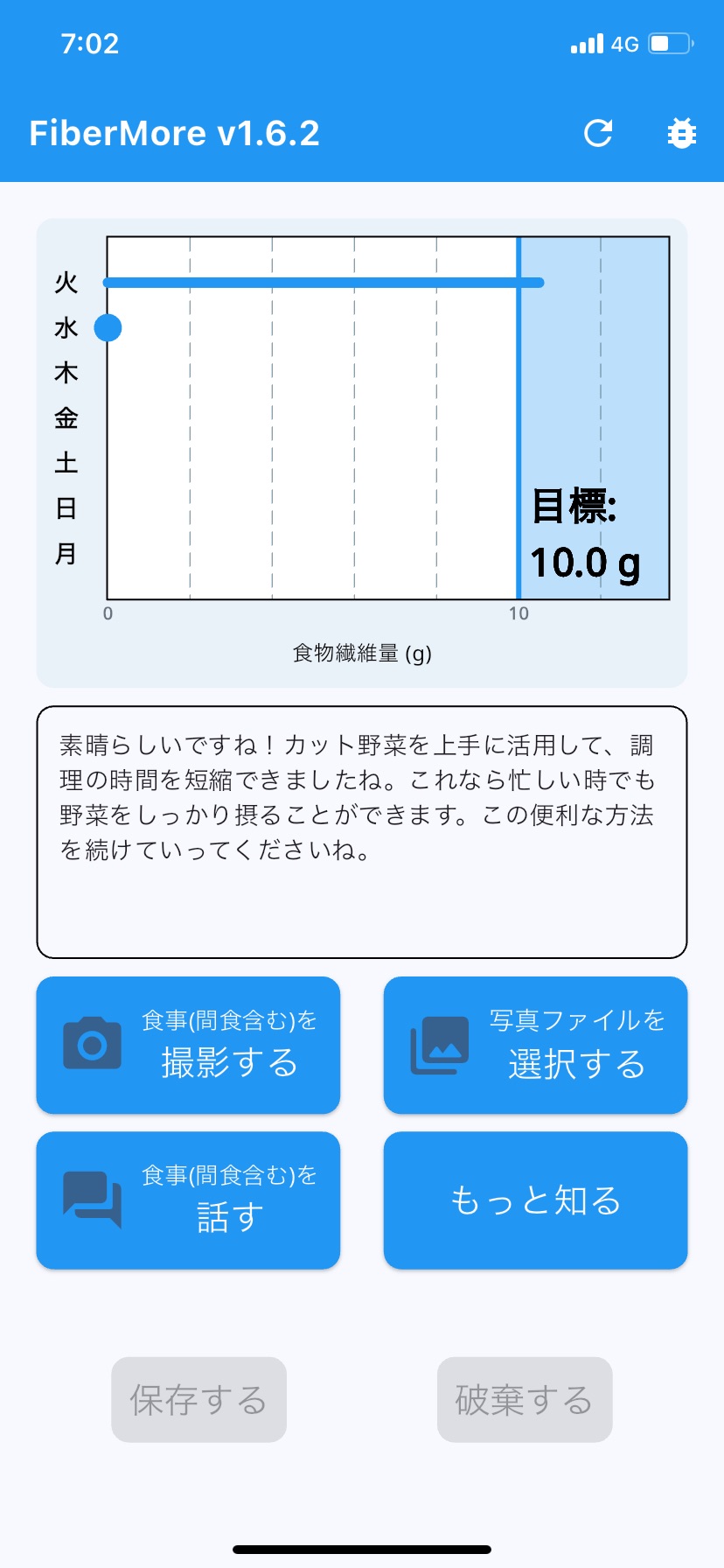


Screenshot 2b: Home screen of FiberMore app with encouragement on implementing solution. (English)

Screenshot 2a: Home screen of FiberMore app with encouragement on implementing solution. (Japanese)

**Step 2: Meal logging and access to information about dietary fiber**

Study participants log meals throughout the day via “Snap a meal” or/and “Chat a meal” functions on the app. They are able to view the progress towards reaching the daily fiber goal via progress bars on the Home screen. For each meal logged, the fiber amount will be displayed on the Home screen, along with the feedback message regarding the fiber-energy ratio of the meal scaled to the target daily fiber and energy intake (Screenshot 3a, 3b).


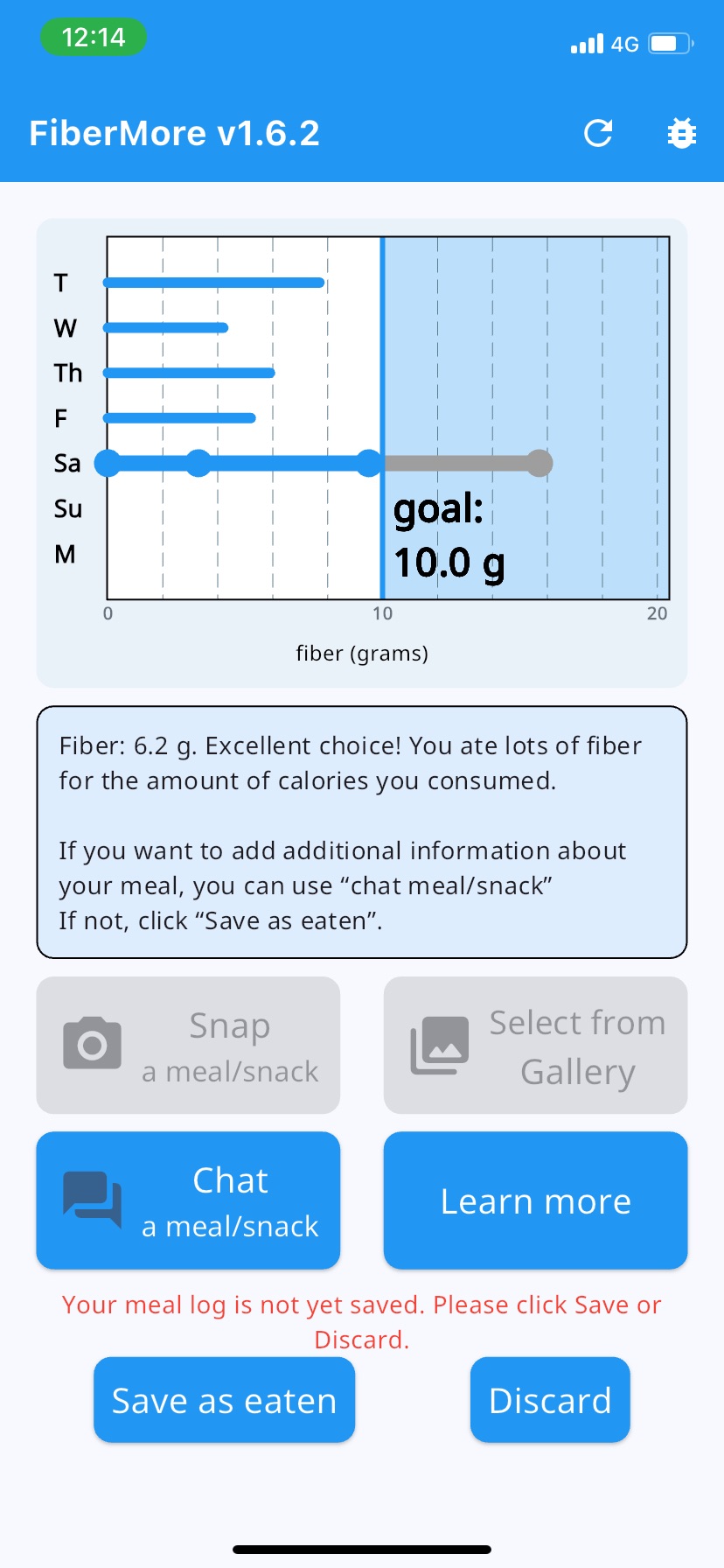


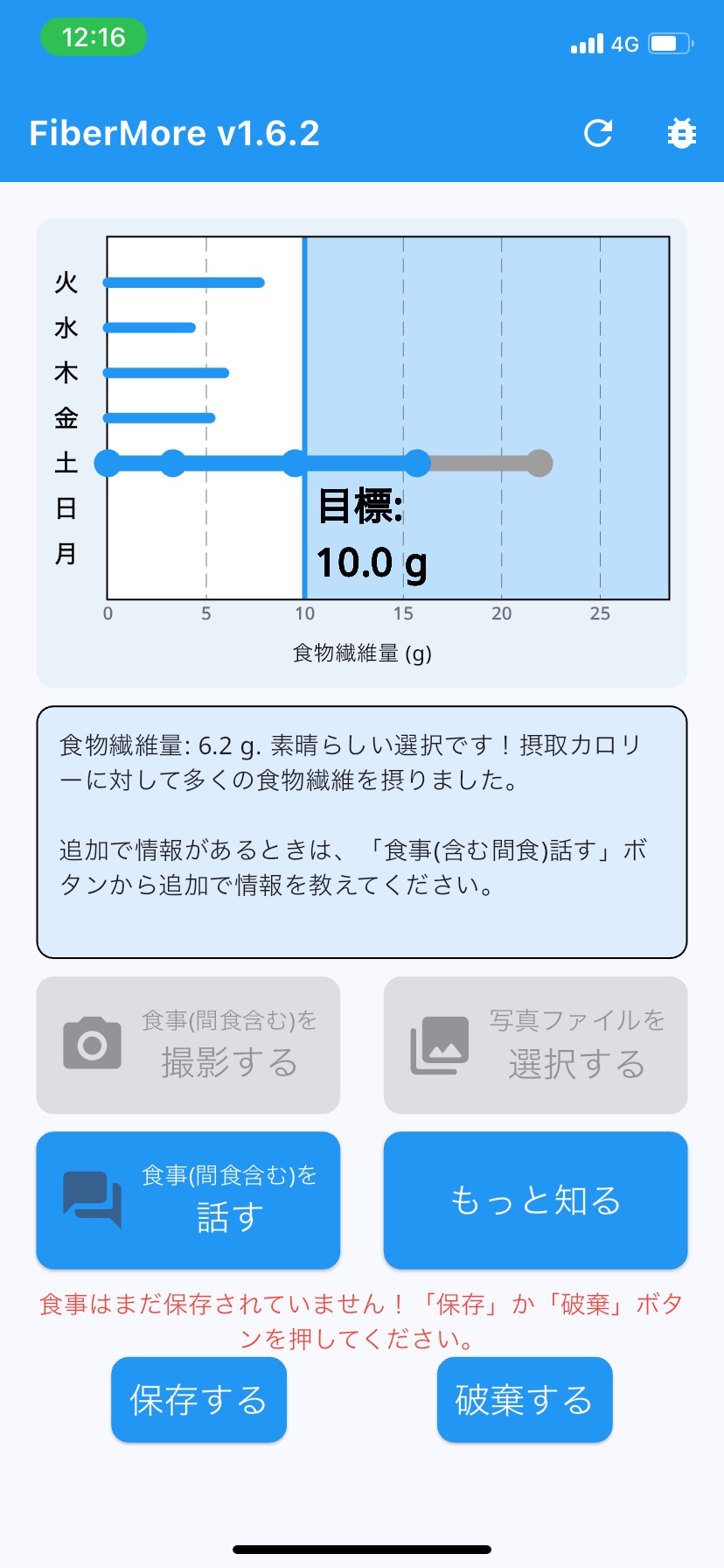


Screenshot 3b: Home screen of FiberMore app with feedback on fiber intake (English)

Screenshot 3a: Home screen of FiberMore app with feedback on fiber intake (Japanese)

Participants can also use the “Learn more” chatbot anytime to ask questions about dietary fiber (Screenshot 4a, 4b).


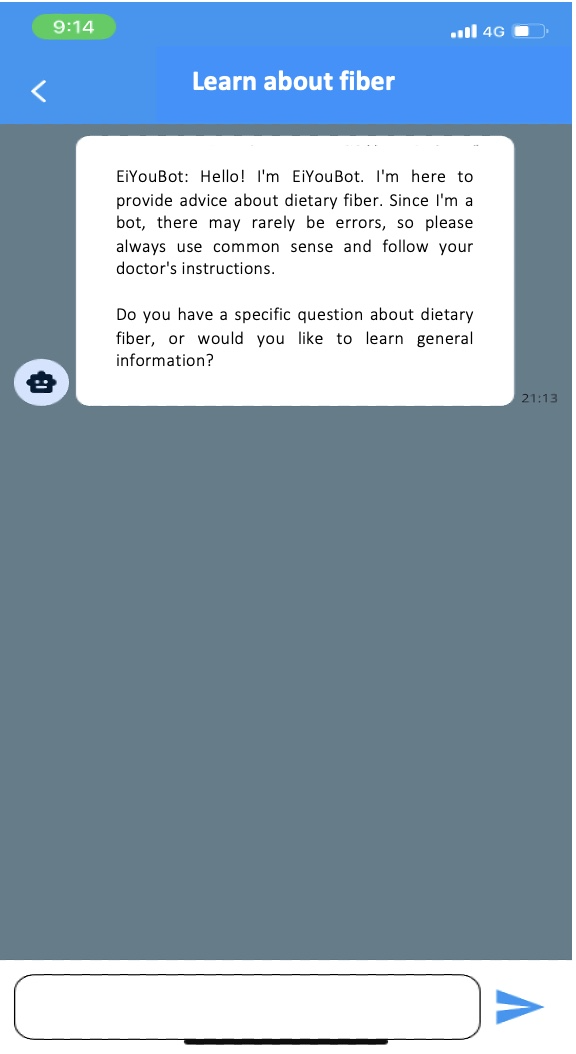


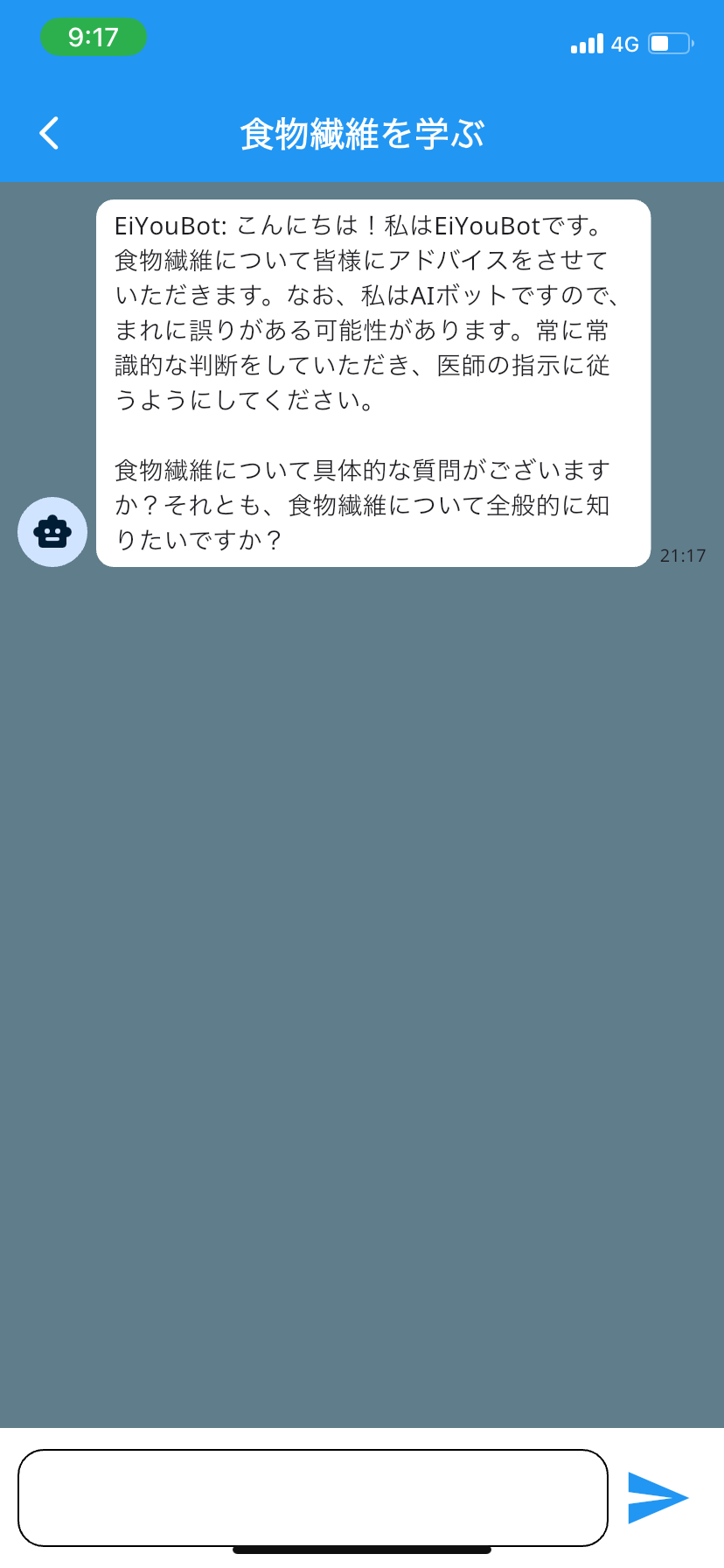


Screenshot 4a: “Learn more” chatbot (Japanese)

Screenshot 4b: “Learn more” chatbot (English)

Screenshot 4b: “Learn more” chatbot

**Step 3: Midweek Checking-In (Day 4)**

On Day 4 from 11AM onwards, when study participants open the app, a pop-up chat will appear with a message from EiYouBot: “We should discuss how you feel about how things are going. Do you have time now?” (Screenshot 5a, 5b).

Participant can choose to respond to the bot message immediately or at a later time, by selecting either “Do now” or “Later” buttons that appear. If study participants click “Do Now”, it triggers the chatbot to engage the user in a discussion on whether the efforts to increase dietary fiber intake is enjoyable, just okay, or not very enjoyable. If participant click “Later”, the message box will appear to allow for delayed engagement later in the day.


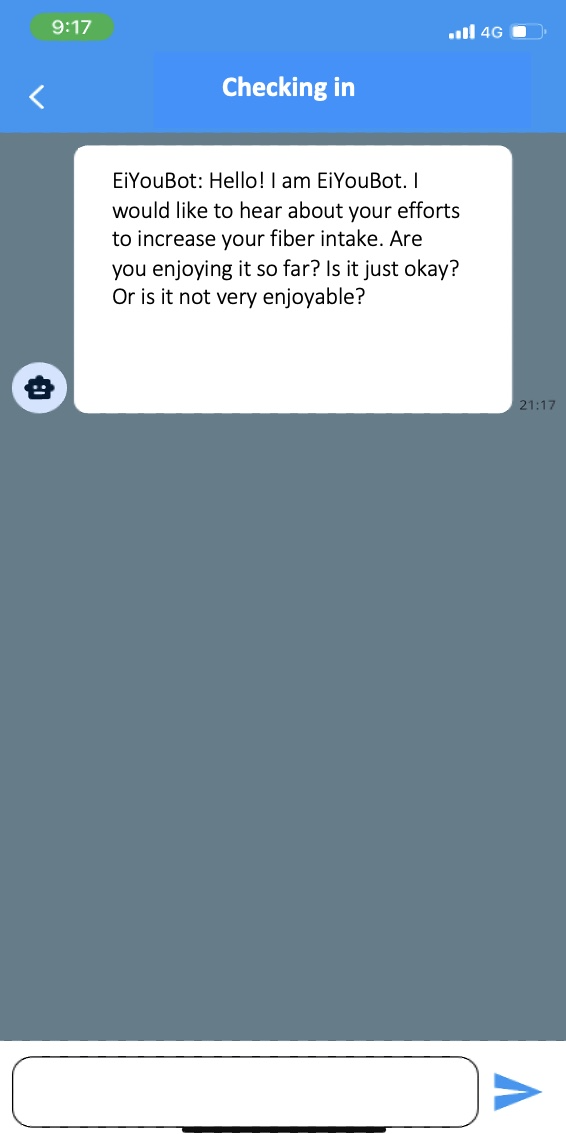


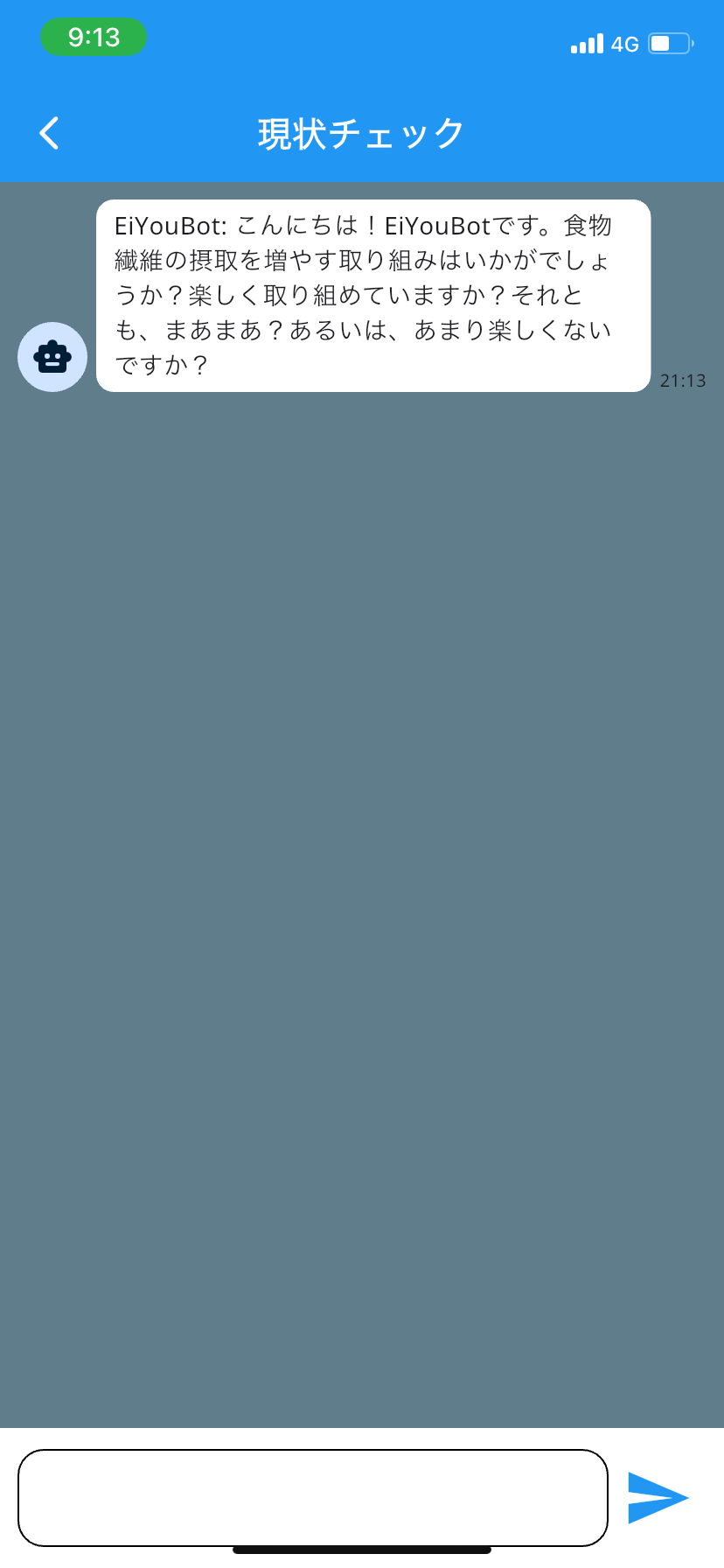


Screenshot 5b: “Checking-in” chatbot (English)

Screenshot 5a: “Checking-in” chatbot

(Japanese)

**Step 4: Weekly barrier, solutions, and goal setting**

7 days after the day of official study enrollment visit, a notification from the app will be sent to the user’s phone at 11AM as a reminder for study participants to set a new weekly barrier and coping strategy (solution) towards eating more fiber (Screenshot 6a, 6b), as well as set a new weekly fiber goal with EiYouBot (Screenshot 7a, 7b). Participant can choose to respond to the bot message immediately or at a later time. If no input on the new week’s barrier and solution are provided by midnight of that day, the app will automatically carry over the previous week’s barrier and solution to the current week. If a participant does not perform goal setting by midnight of the day, the app automatically assigns a goal based on the goal setting algorithm of FiberMore (Table 2).


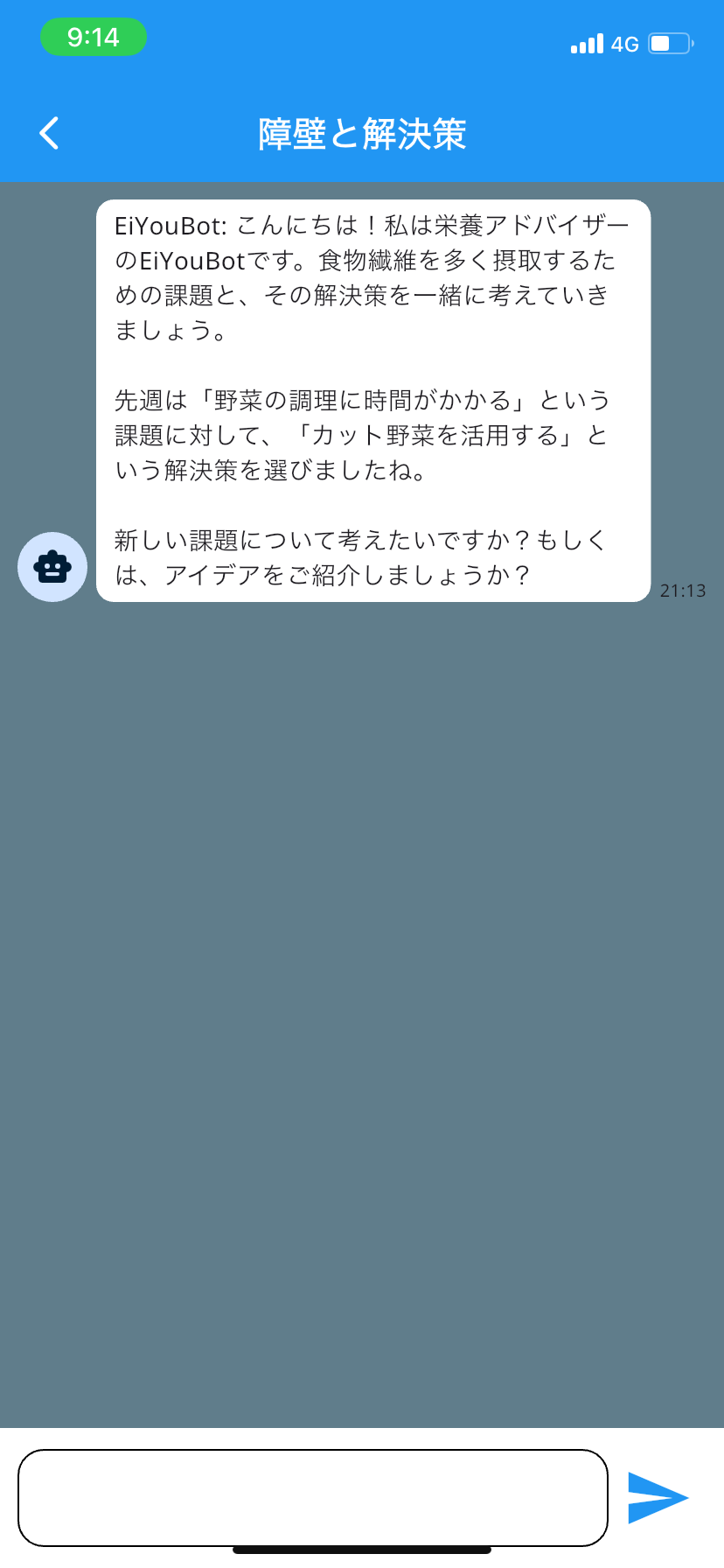


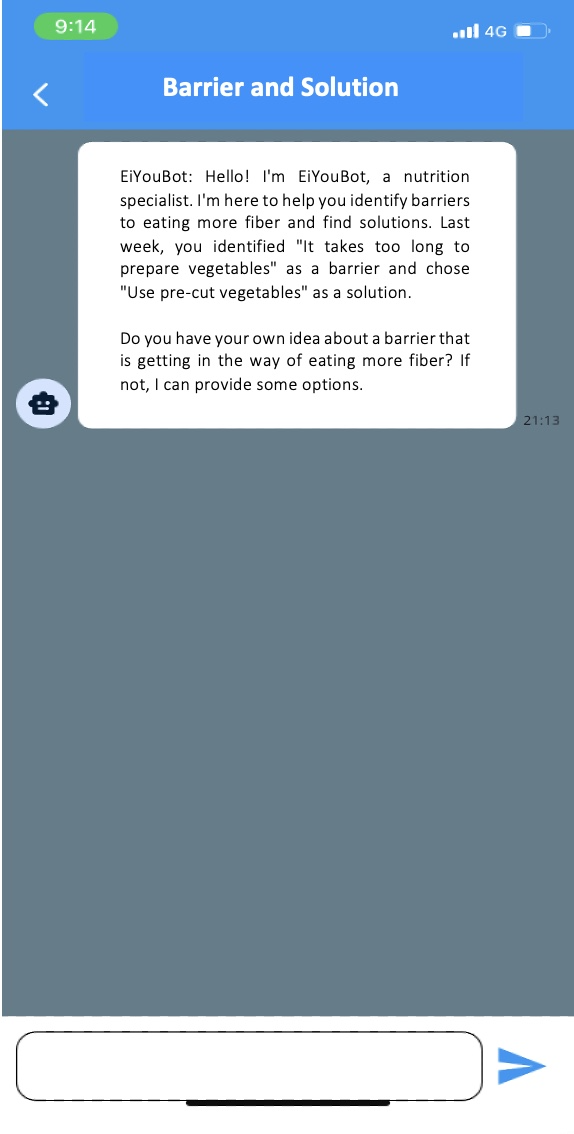


Screenshot 6a: “Barrier and solution setting” chatbot (Japanese)

Screenshot 6b: “Barrier and solution setting” chatbot (English)


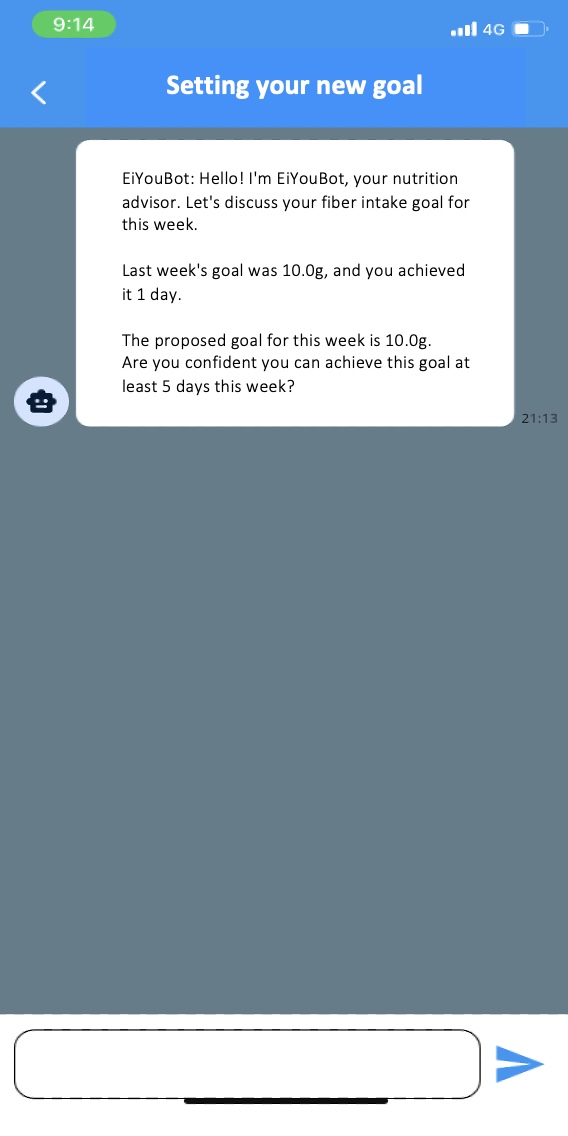


**
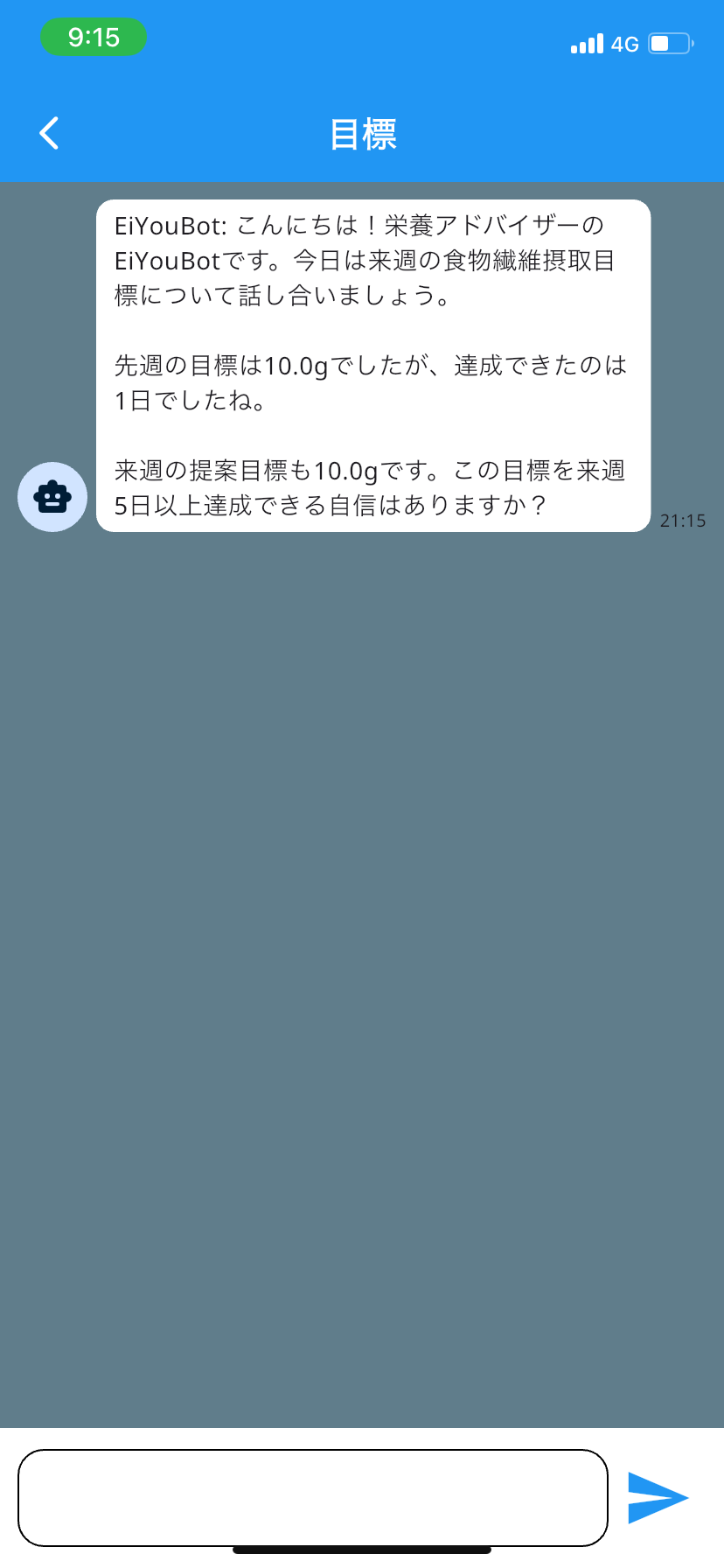
**

Screenshot 7b: “Goal setting” chatbot (English)

Screenshot 7a: “Goal setting” chatbot (Japanese)
